# Microbially-Induced Carbonate Precipitation in Low pH Cement; Potential for Self-Healing in Radioactive Waste Geodisposal Systems

**DOI:** 10.1021/acsomega.5c04312

**Published:** 2025-11-19

**Authors:** Ananya Singh, Natalie Byrd, Dirk Engelberg, Christopher Boothman, Samuel Shaw, Katherine Morris, Jonathan R. Lloyd

**Affiliations:** † Department of Earth and Environmental Science, Radioactive Waste Disposal and Environmental Remediation (RADER) National Nuclear User Facility and Williamson Research Centre, 5292 The University of Manchester, Manchester M13 9PL, U.K.; ‡ Metallurgy and Corrosion, Department of Materials, The University of Manchester, Manchester M13 9PL, U.K.; § Manchester Institute of Biotechnology, The University of Manchester, Manchester M13 9PL, U.K.

## Abstract

In a geological disposal facility for radioactive waste, groundwater interacts with engineered cement barriers used for waste encapsulation, as a backfill material, and for construction purposes. This study investigates microbial interactions with low-pH cement (pH 10.5 and 11) in synthetic groundwater under anoxic conditions. Microcosm experiments used alkaliphilic microbes obtained from a high-pH lime kiln site. These were incubated with low-pH cement tablets in synthetic Oxford clay porewater with varying levels of electron donors (lactate or hydrogen) or electron acceptors (nitrate). In higher-carbon systems supplemented with lactate, reduction of nitrate was noted over six months, decreasing pH (10.0 to 8.8), presumably due to carbonic acid production. Calcium concentrations also dropped (9.2 to 4.7 mM). SEM-EDS and μ-XCT analyses confirmed that calcium carbonate precipitates had healed cracks and reduced porosity in the cement tablets. However, in the low-carbon system, microbial activity was minimal, leading to smaller pH changes (from 10.0 to 9.4) and greater leaching of Ca^2+^ and Mg^2+^ from the cement. This indicates that less carbonate was produced, and the reduction in pore size over six months was smaller compared to the high-carbon system. 16S rRNA gene sequencing revealed an increased abundance of alkaliphilic nitrate-reducing bacteria, including *Anaerobacillus* species, which were enriched in high-carbon treatments. Overall, findings suggest that in low-carbon environments, cement will gradually leach cations. By contrast, elevated organic carbon levels (e.g., from cementitious waste) can stimulate microbial activity, enhancing calcite precipitation, crack healing, and pore-clogging, which could improve cement barrier performance and limit contaminant migration.

## Graphical abstract

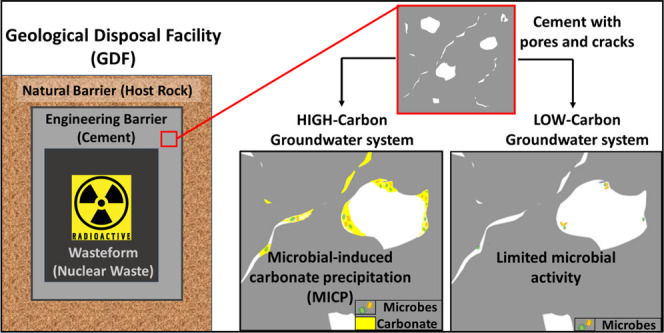

## 1 Introduction

Low-level waste (LLW) and intermediate-level waste (ILW) comprise approximately 97% of the global nuclear waste inventory by volume.^1^ These wastes are predominantly produced by nuclear fuel cycle operations, medical science, research, and the military (e.g., from nuclear-powered submarines).^2^ Early nuclear nations (e.g., France, Russia, the United States, and the United Kingdom) have produced the largest volumes of L/ILW due to decommissioning of extensive legacy facilities.^3^ In the UK, these nuclear wastes are stored in highly monitored and maintained artificial ponds. However, this is not a permanent solution. Recent forecasts indicate at least a 4.5 million tons increase in radioactive waste inventory by the next century in the UK, with around 4.4 million tons designated as L/ILW.^1,4^

To address this problem, the International Atomic Energy Agency has established accepted guidelines for the disposal of radioactive waste worldwide, focusing on nuclear waste management and the safe isolation of waste radionuclides. For LLW, near-surface repositories have been considered, while for ILW, specially designed deep geological disposal facilities (GDFs). Both facilities use a multibarrier approach to prevent contaminant migration.^5,6^ These barriers include the conditioned waste form (e.g., solidified or compacted waste in iron or steel containers), a geotechnical barrier/backfill material (e.g., bentonite and cement), and a geological barrier (the host rock, e.g., lower strength sedimentary rock or clay).^7^

Cement is widely used in radioactive waste disposal facilities as a construction material for structural components such as vaults and tunnels, geotechnical backfill, and an encapsulant.^8,9^ Cement formulations can be tailored to achieve these specific performance outcomes. For example, Portland cement-based concretes are the most common construction materials in repositories due to their low permeability and high strength, which are essential to prevent water ingress and structural collapse during the long preclosure period.^9^ In contrast, cement used in geotechnical backfill within the engineered barrier system can be tailored to allow for controlled permeability, which helps release gases—a critical aspect of the geological disposal facility (GDF) safety design.^8^ Another valuable function of Ordinary Portland Cement (OPC) is its ability to chemically immobilize radionuclides through interactions with various cement phases.^8–10^ The high pH (>12) of OPC offers benefits such as enhanced mechanical performance and reduced radionuclide mobility in leachate environments.^9,10^ However, this same high pH can pose challenges. It may corrode metals like Al, Zn, and Mg, leading to hydrogen gas generation.^11,12^ Moreover, the hyper-alkaline leachates can create a chemically disturbed zone (CDZ) extending from cement into surrounding materials.^13,14^ Bentonite, used as a swelling clay barrier for high-level and intermediate-level waste (H/ILW) due to its low permeability and ion exchange properties,^10,15^ is particularly vulnerable in CDZ. High-pH cement leachates can impair the swelling and sorptive capacities of bentonites, potentially undermining their effectiveness as a barrier.^8,16–18^

The potential negative impacts of the hyper-alkaline conditions created by high-pH cement in GDFs have highlighted the need for improved cement formulations.^8,16,17^ New cement formulations are necessary to optimize cement performance by reducing its hyper-alkaline nature while maintaining the benefits of cement as a chemical barrier and preserving the structural integrity of the repository. To address this, alternative “low-pH cement” formulations have been developed for use in long-term radioactive waste disposal systems.^17,19–22^ These formulations are specially designed to reduce the chemical disturbance in repositories where a less alkaline (pH < 11) and less corrosive environment is required.^17,18^ This reduced pH is achieved by lowering the amount of OPC and substituting it with materials such as fly ash, blast furnace slag (BFS), silica fume (SF), or nonpozzolanic flour.^16^ This leads to a cement matrix with little or no free portlandite and comprises calcium silicate hydrate (C–S–H) gel with a low Ca/Si ratio (≤0.8), which lowers the buffering capacity and improves mechanical stability.^16,20^ A superplasticizer is also added to slow the setting rate, increasing the “workability” of low pH-cement.^16^ A reference low-pH formulation known as CEBAMA was developed by VTT (Valtion Teknillinen Tutkimuskeskus) in Finland as part of a European project.^17,18^ The CEBAMA formulation is now considered a strong candidate for use in disposal environments where exposure to high pH conditions must be limited.^18^ Due to its relevance and growing acceptance, this formulation was adopted in the present study.

In addition to CEBAMA, several other low-pH cement formulations have been proposed in the literature, including silica-fume blended OPC,^23,24^ calcium sulfoaluminate (CSA) cement,^25,26^ and limestone calcined clay cement (LC3).^27^ These formulations typically achieve pH values in the range 10–11, comparable to the CEBAMA mix. However, CEBAMA demonstrates a well-documented balance of low pH, structural performance, and chemical compatibility, which makes it a useful option for GDFs.^28–30^ However, understanding the long-term evolution of low-pH cement is important for developing a robust environmental safety case for a GDF. This requires evaluation of the effects of both abiotic and biotic factors within the GDF system. The influence of abiotic factors—such as groundwater, gas phase, and soil components—has been extensively studied on the chemo-mechanical properties and evolution of low-pH cement.^28,31^ Cement types, such as CSA and LC3, have also been evaluated for carbonation resistance, primarily in reinforced concrete applications where carbonation is detrimental due to its impact on steel corrosion.^23–27,32^ In unreinforced systems like waste form encapsulation and backfill of a geological repository, however, carbonation may be beneficial, as it can help seal pores and enhance long-term containment. Importantly, under the anaerobic conditions expected in GDFs, abiotic calcium carbonate formation is limited, pointing to the potential role of microbes in mediating mineralization. Despite the extreme conditions expected in GDFs, alkaliphilic and anaerobic microbial colonization is still anticipated.^33–36^ Therefore, along with abiotic factors, studying biotic influences is necessary to ensure the long-term safety and performance of low-pH cement systems in radioactive waste disposal. This opens an important research area that requires investigation: microbial–cement interactions under geological repository-relevant conditions.

While microbial activity is often associated with adverse impacts on cement, such as biocorrosion,^37–39^ it can also offer potentially beneficial effects on long-term cement performance in a GDF setting. One such beneficial process could be microbial metabolism that produces carbonate through the oxidation of organic compounds. These carbonates can react with calcium and magnesium ion, leached from the cement, leading to oversaturation and precipitation of Ca- and Mg-carbonate phases within cracks and pore spaces.^40–44^ This process, known as microbially induced carbonate precipitation (MICP), has been shown to heal microcracks and fill pores in high-pH OPC systems.^40–44^ Studies have also reported significant improvements in the mechanical properties of concrete following MICP-based healing, including an increase in compressive (15%)^45^ and flexural strength (28–50%)^46^ in a month. However, these findings are largely derived from systems operating under aerobic conditions. In contrast, many recent studies have confirmed microbial colonization of extreme environments, including anaerobic, alkaliphilic environments.^36^ These environments are relevant to the extreme chemical environment expected in cementitious GDFs.^34–36,47–51^ Despite this potential, the influence of MICP in low-pH cement under geological repository-relevant (anaerobic and alkaliphilic) conditions remains largely unexplored. Given the potential for microbial activity to impact cements in a GDF, either through cement biodegradation, or indeed biocementation leading to reduced porosity and decreased radionuclide transport, understanding how microbial metabolism behaves in low-pH cement systems—particularly under varying groundwater–carbon availability—is important. Addressing this knowledge gap is a primary objective of the present study.

This work aims to quantify the impact of microbial metabolism on cement integrity under anoxic, GDF representative conditions, recognizing that such microbial activities could have beneficial or detrimental effects. Postclosure, repositories are expected to become saturated with groundwater infiltration, which can potentially supply nutrients that support microbial growth. These nutrients may include dissolved salts (e.g., phosphate and the electron acceptor sulfate) and organic carbon derived from the degradation of radioactive waste components, such as cellulose.^31,34,43^ The availability of organic carbon is expected to play a key role in controlling microbial proliferation by serving as a substrate for metabolism. In this study, we examined the influence of varying carbon loadings on the rate and extent of MICP, while also evaluating any evidence of cement degradation. This was assessed in a series of model systems designed to simulate GDF–relevant groundwater conditions, with particular focus on interactions involving low-pH cement chemistry.

## 2 Materials and Methods

Microcosm experiments were set up containing CEBAMA low-pH cement tablets, artificial groundwater, and nitrate as electron acceptors and incubated for six months at 20 °C. High-carbon (lactate) and low-carbon (yeast extract, supplemented with hydrogen) conditions were compared in the context of MICP. All systems were analyzed using geochemical methods (ion chromatography (IC), inductively coupled plasma atomic emission spectroscopy (ICP-AES), and pH), mineralogical analyses (SEM-EDS, FT-IR, and XCT), and microbial community analysis (16S rRNA gene sequencing). Geochemical modeling was conducted using PHREEQC^52^ in conjunction with the Thermochimie database^53^ (version 9b) to support experimental planning and data interpretation.

### 2.1 Groundwater composition

Synthetic groundwater was prepared, containing CaCl_2_, 1.21 g/L; MgCl_2_·6H_2_O, 1.62 g/L; NaCl, 18.3 g/L; KCl, 0.627 g/L; NaHCO_3_, 0.428 g/L; Na_2_SO_4_, 4.99 g/L; and Na_2_SiO_3_·5H_2_O, 0.023 g/L, informed by pore waters data from relevant lower strength sedimentary rocks.^54^ The starting pH of synthetic groundwater was 8.3.

### 2.2 Cement Tablets

Cement tablets were prepared using the low-pH CEBAMA reference mix.^17^ The mix included: low alkaline OPC (CEM I 42.5R), 954 g; densified SF, 1000 g; BFS, 590 g, and naphthalenesulfonate superplasticizer, 11.24 mL to achieve the desired workability, with a water/solid ratio of 0.6 by weight. The cement paste was cast in small tablet molds (6 mm in height and 10 mm in diameter) and air-dried for 28 days before demolding. Prior to use in the microcosms, the cement tablets were rinsed with deionized water and gently agitated to remove any surface deposits. They were then equilibrated for 1 week in deionized water until the pH was stabilized to promote cement hydration reactions and facilitate the formation of the calcium silicate hydrate (C–S–H) phase. After this, the cement tablets were added to microcosm experiments without any further treatment.

### 2.3 Inoculum Sediment Sampling

Sediment slurry (pH 11.7) was collected from a depth of ∼20 cm at a well-characterized, calcite-rich legacy lime working site in Harpur Hill, Derbyshire, UK.^29^ The sample was stored at 10 °C in the dark and used within 8 weeks of collection. This high-pH sediment is enriched in calcium and silicate, making it relevant to GDF systems.^49,55^ The presence of potential electron acceptors and volatile fatty acids (VFAs) was analyzed by using IC.

### 2.4 Microcosm Setup

Microcosms amended with different treatments were set up containing: (i) no added carbon, (ii) low-carbon, with yeast extract (15 mg/L), and a 100% hydrogen headspace as electron donor, and (iii) high-carbon with lactate (15 mM). These anaerobic microcosms were set up in triplicate and contained 30 mL of synthetic groundwater (approximately 20 mL of headspace), the respective carbon source/electron donor, 5 g of Harpur Hill sediment inoculum, nitrate (∼20 mM) as an electron acceptor, and four cement tablets (5.5 g each). The headspace of the no-added carbon and high-carbon systems was replaced with nitrogen prior to inoculation. The headspace of the low-carbon system was replaced with hydrogen as an additional GDF-relevant electron donor that is expected to be released through the anaerobic corrosion of electropositive metals such as steel in the repository.^11,12^ Parallel experiments with no nitrate were also performed. The cement tablets and sterile groundwater were added to sterilized serum bottles and stored in the dark for 3 days until the pH had stabilized (pH 10.3), prior to inoculation with sediment. Following pH stabilization, the sediment inoculum, carbon source, and nitrate (filter sterilized) were added to the relevant bottles. The microcosms were incubated at 20 °C in the dark for six months, and approximately 1 mL of slurry was withdrawn periodically using an anaerobic sampling technique for geochemical and microbial analysis. One cement tablet was removed in anaerobic conditions from each of the treatments after one, two, three, and six months for characterization by SEM-EDS (mineralogical) and μ-XCT (structural). (Supplementary Table S1 summarizes the system and different treatments in the microcosm experiments.)

### 2.5 Geochemical Analysis

The pH was measured using a Denver Instrumental digital meter and a Fisherbrand FB68801 electrode, routinely calibrated with pH 7, 10, and 12 buffers (Thermo Fisher Scientific). The samples were centrifuged for 10 min at 14,000*g*, and supernatants were filtered before analysis by IC and ICP-AES. A Dionex ICS5000 IC instrument was used to measure the concentration of anionic species (selected VFAs, nitrate, nitrite, and sulfate). Supernatants were diluted 200-fold in deionized water prior to analysis. ICP-AES measurements of calcium and magnesium were performed by using a PerkinElmer Optima 5300 ICP-AES instrument. Filtered supernatants were diluted in a 2% (v/v) nitric acid solution before analysis.

### 2.6 Mineralogical Analysis and Imaging

#### 2.6.1 X-ray Diffraction

Prior to use, the CEBAMA reference mix and the sediment inoculum were characterized by XRD using a Bruker D2 Phaser diffractometer with a Cu Kα X-ray source (wavelength = 1.54 Å).

#### 2.6.2 Scanning Electron Microscopy and Energy-Dispersive X-ray Spectroscopy

Under anaerobic conditions, cement tablets were carefully extracted from the microcosm bottles by using a sterilized steel wire and gently rinsed with deionized water to remove any loose particles from the surface. Before scanning electron microscopy (SEM) imaging, whole cement tablet specimens (0.6 × 1.0 cm) were air-dried and mounted onto the SEM sample holder. Analysis was performed on an FEI Quanta 650 FEG­(E)­SEM instrument operated at 15 kV in a low-vacuum (60 bar). Corresponding EDS spectra of the mineralized carbonate deposits were collected from all samples. Thin section samples of the cement tablets were prepared by resin-embedding, cutting, and polishing the pieces for EDS mapping. The mapping of these samples was performed over 500 pixels under a low vacuum (10 kV and 60 bar).

#### 2.6.3 Fourier Transform Infrared Spectroscopy

Fourier transform infrared (FTIR) spectra were acquired by using a PerkinElmer Spectrum 100 instrument equipped with a Universal Attenuated Total Reflectance (ATR) accessory. The ATR setup consisted of a diamond/ZnSe crystal (single reflection), a top plate, and a pressure arm with a force indicator. Spectra were recorded over a range of 4000 to 650 cm^–1^, with a resolution of 4 cm^–1^, using 16 scans and triplicate analyses for accuracy. A clean ATR diamond/ZnSe crystal was used to record the background spectra prior to sample analysis.

#### 2.6.4 Micro X-ray Computed Tomography

Cement samples were characterized by μ-XCT using a Zeiss VersaXRM620-DCT (Zeiss Metrology, Oberkocken, Germany). The Scout and Scan application was applied, with an optical magnification of 0.4× times, giving a pixel size of 5 μm with a 3.5 s exposure time for each X-ray image. 80 kV with a beam current of 87.48 μA was used for all scans. A large data set of 3201 slices was obtained for each scan, with 3D volume reconstruction performed after correcting for center shift and beam hardening. The images were saved as TXM files and segmented using the Avizo 2020.2 software package (Thermo Fisher Scientific). Segmentation of pores, surface carbonates, and cement phases was conducted by using the histogram range tool and interactive thresholding techniques. Label analysis was utilized to determine the pore diameters. Pores were classified into four groups based on their equivalent diameters using a sieve analysis tool (100–200 μm, 200–300 μm, 300–400 μm, and 400–800 μm). Identical processing methods were applied consistently across all sample data (Further detailed protocols are given in Supporting Information Section 5).

### 2.7 Microbial Community Analysis

#### 2.7.1 DNA Extraction

A DNeasy PowerLyzer PowerSoil Kit (Qiagen, Manchester, United Kingdom) was used for extracting DNA from 800 μL aliquots of the sediment slurry. The extracted 16S rRNA gene fragments were amplified using the Polymerase Chain Reaction (PCR) using 8F (5′-AGAGTTTGATCCTGGCTCAG-3′) primers and 1492R (5′-TACGGYTACCTTGTTACGACTT-3′) primers.^56^ After amplification, the DNA was stained and subjected to agarose gel electrophoresis. Stained DNA was observed under UV light, and the target ∼1500 base pair products were identified by comparison with a DNA ladder of varying fragment length. Experimental controls were performed to check for reagent contamination.

#### 2.7.2 16S rRNA Gene Sequencing

An Illumina MiSeq platform (Illumina, San Diego, Ca, USA) was used to sequence the 16S rRNA gene PCR amplicons targeting the V4 hypervariable region (forward primer 515F, 5′-GTGYCAGCMGCCGCGGTAA-3′; reverse primer, 806R, 5′-GGACTACHVGGGTWTCTAAT-3′) for 2 × 250-bp paired-end sequencing^57,58^ (Illumina). PCR amplification was performed using a Roche FastStrat High Fidelity PCR System (Roche Diagnostics Ltd., Burgess Hill, UK) in 50 μL reaction volumes under the following conditions: initial denaturation at 95 °C for 2 min, followed by 36 cycles of 95 °C for 30 s, 55 °C for 30 s, 77 °C for 1 min, and a final extension step of 5 min at 72 °C. PCR products were purified and normalized to approximately 20 ng per sample using the SequalPrep Normalization Kit (Thermo Fisher Scientific, Loughborough, UK). The normalized PCR amplicons from all samples were pooled in equimolar ratios. The sequencing run was performed with a 4 pM sample library, spiked with 4 pM PhiX to a final concentration of 10%, following the method outlined by Schloss and Kozich.^59^ Raw sequences were demultiplexed based on barcodes, allowing up to one mismatch, using a sequencing pipeline. Quality control and trimming were conducted using Cutadapt, FastQC, and Sickle. Error correction for MiSeq reads was performed using SPAdes, and forward and reverse reads were merged into full-length sequences with Pandaseq. Chimeric sequences were removed using ChimeraSlayer, and operational taxonomic units (OTUs) were clustered using UPARSE at 97% similarity. Singletons were excluded, and rarefaction analysis was carried out on the detected OTUs by using Qiime. Taxonomic classification was done with the RDP classifier, following methods by Caporaso (2011) and Kozich (2013). This process was performed for all of the treatments after days 3, 114, and 164.

## 3 Results and Discussion

The mineralogy of the CEMABA mix and its major components used in the formula to prepare cement tablets were first analyzed using XRD, revealing a predominantly calcite-rich composition (Supplementary Figures S1–S3). Additionally, XRD analysis of the Harpur Hill sediment used as a microbial inoculum also confirmed to have a calcite-rich mineralogy (Supplementary Figure S4). Furthermore, IC results (Supplementary Table S2) indicated the presence of very low concentrations of VFAs and potential electron acceptors in the Harpur Hill inoculum.

Anaerobic microcosms were designed to simulate low pH cement (pH ∼10) conditions relevant to a late postclosure cementitious GDF, with exposure periods over 6 months. Three different treatments were used to reflect a range of organic carbon levels: high-carbon (15 mM lactate), low-carbon (15 mg/L yeast extract, with 100% H_2_ headspace), and no-carbon. Microcosms were set up with nitrate (20 mM) added as an electron acceptor, present in some nuclear waste streams,^49^ alongside no-nitrate controls for each treatment. Two additional abiotic controls were set up: (1) microcosms with autoclaved Harpur Hill sediment inoculum to rule out nonbiological deposition of carbonate phases (Supporting Information Section 8) and (2) microcosms without inoculum to monitor chemical cement leaching under parallel conditions to the microbially active experiments (Supporting Information Section 9). Results are presented in the following order: high-carbon, low-carbon, no-carbon, and their respective no-nitrate control systems, providing a comparative overview of the effects of varying carbon availability and electron acceptor presence on cement alteration processes. Results from the additional control experiments are presented in the Supporting Information sections.

### 3.1 Geochemical Results

The high carbon systems were set up to reflect potential niches within a GDF where elevated organic carbon levels may be present, e.g., from cellulose degradation products and organic decontaminants from the waste. In these high carbon systems, lactate concentrations decreased from 17.1 ± 1.8 mM to 2.35 ± 0.91 mM by the experimental end-point at 168 days, while acetate concentrations increased from 0.0 mM to 7.79 ± 0.11 mM (Figure 1A). The decrease in lactate concentration was accompanied by a decrease in nitrate concentration from 19.9 ± 0.6 mM to 0.02 ± 0.01 mM and an increase in nitrite concentration from 0.0 to 14.1 ± 0.7 mM (Figure 1B). These changes indicate that nitrate reduction, predominantly to nitrite, coupled to lactate oxidation, was occurring in the high-carbon system, consistent with other reported high pH, nitrate-reducing microcosm systems in the literature.^34,47,49,50^ Maximal rates of acidification were also observed (Figure 1C), likely due to nitrate reducers catalyzing the partial oxidation of lactate to acetate and carbon dioxide, which dissolves to form carbonic acid.^50,60^ Between pH 9–10, carbonic acid equilibrates in solution to produce carbonate (CO_3_^2–^) and bicarbonate (HCO_3_^–^) ions, which can contribute to the oversaturation and precipitation of carbonate mineral phases when Ca^2+^ and Mg^2+^ ions are present. Indeed, Ca^2+^ and Mg^2+^ were included in the artificial groundwater at concentrations of 10.8 and 8.1 mM, respectively. ICP-AES analysis of this system suggested a decrease in Ca^2+^ and Mg^2+^ ion concentrations in solution, from 9.2 ± 0.3 mM to 4.7 ± 0.4 mM and 8.2 ± 1.4 to 5.8 ± 0.5 mM, respectively, presumably due to precipitation of Ca/Mg-carbonates. PHREEQC modeling supports this interpretation, as modeling of the experimental end-point solutions from the high-carbon system (Supporting Information Section 4) predicted that Ca and Mg carbonate phases were oversaturated.

**Figure 1:**
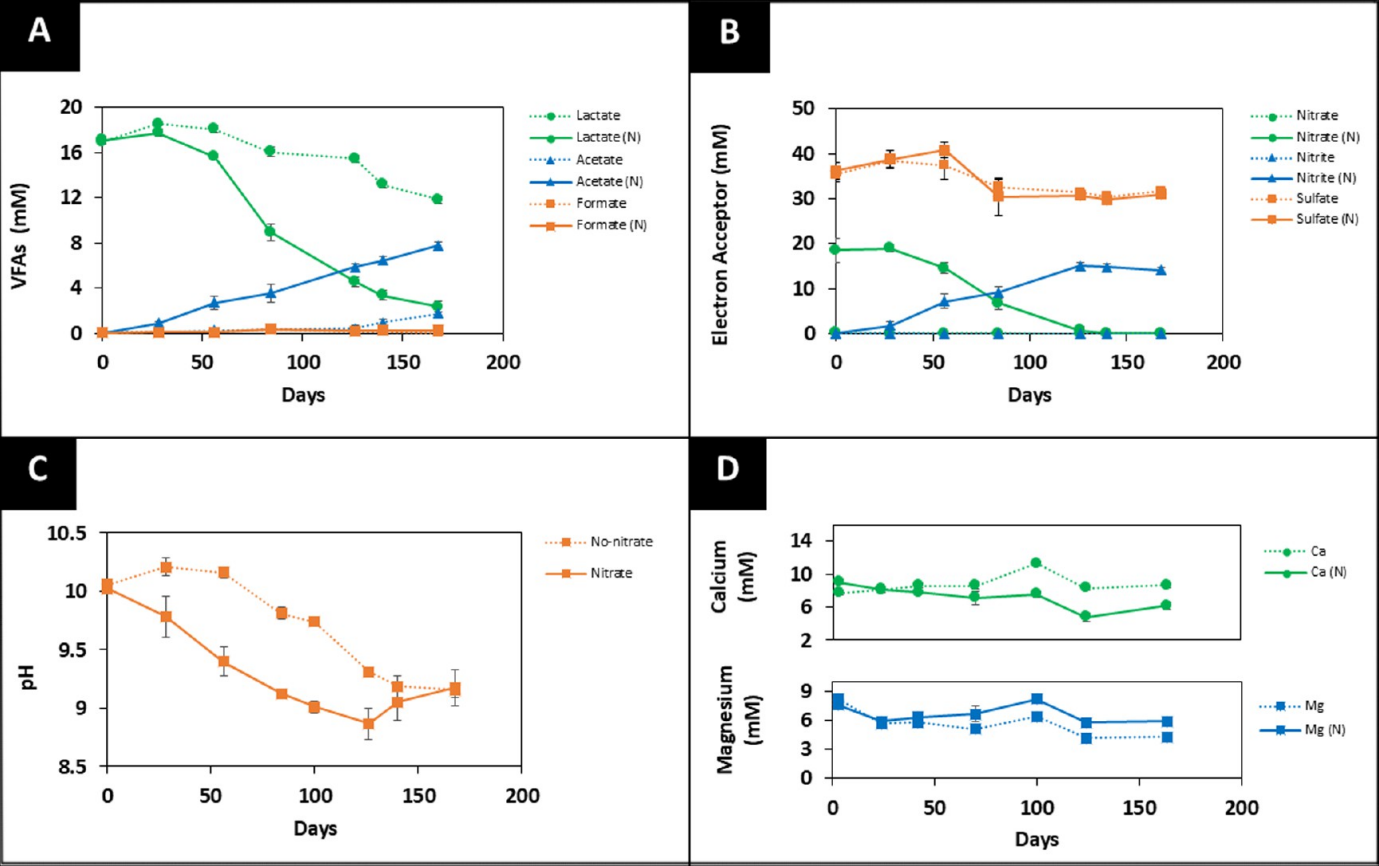
Geochemical data for the high-carbon system. (A) VFA, (B) electron acceptor, (C) pH, and (D) Ca^2+^ and Mg^2+^ concentrations from a 6 month incubated system at 25 °C. “N” is used to represent the system with added nitrate. The dotted lines (······) represent no added nitrate incubations, and the solid lines (______) represent systems with 20 mM nitrate added. The errors shown represent the standard deviation on the triplicate measurements.

In the no-nitrate, high-carbon control, geochemical changes were less pronounced. Over six months, 5.1 mM lactate was consumed, but only a small amount of acetate was produced (Figure 1A; dotted line). Acidification also progressed more slowly in these controls (Figure 1C; dotted line), indicating reduced microbial activity overall. In this system, where electron acceptor concentrations were limited, the modest lactate consumption over six months may be attributed to minor fermentation processes occurring. ICP-AES data for Ca^2+^_(aq)_ revealed a stable concentration of 8.4 ± 0.1 mM throughout the experiment. Aqueous Mg^2+^ levels decreased from 8.20 ± 0.6 mM to 5.1 ± 0.7 mM within the first month and remained stable thereafter in the no-nitrate controls (Figure 1D; dotted line). The reduced microbial activity and lack of Ca^2+^ and Mg^2+^ ion removal in these no nitrate controls further confirm that lactate oxidation coupled to nitrate reduction was driving precipitation of carbonate mineral phases in the high carbon experiments with nitrate as the electron acceptor.

Low-carbon and no-carbon systems showed minimal changes in VFAs (Figures 2A and 3A, respectively) and nitrate (Figures 2B and 3B, respectively) levels in comparison to high-carbon systems over the six-month period. Using H_2_ as an additional electron donor in the low-carbon system did not appear to stimulate the microbial activity. Accordingly, the pH drop from 10.2 to 9.3 (Figures 2C and 3C) was less pronounced in these systems than in the high-carbon system, likely due to the limited supply of organic nutrients, which supported only low levels of microbial activity.

**Figure 2:**
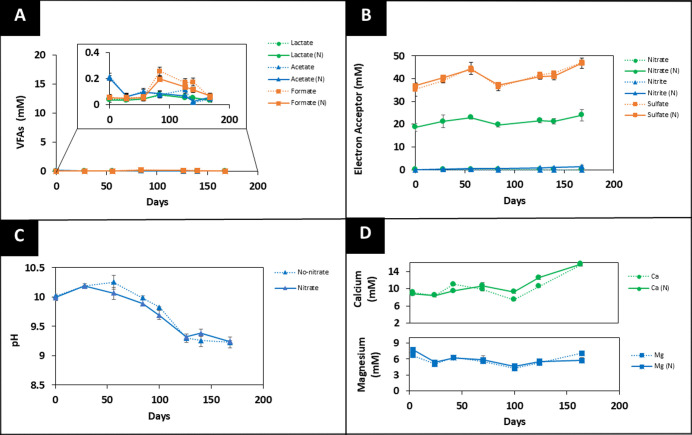
Geochemical data for the low-carbon system. (A) VFA, (B) electron acceptor, (C) pH, and (D) Ca^2+^ and Mg^2+^ concentrations from a 6 month incubated system at 25 °C are presented. (“N” is used to represent the system with added nitrate). The dotted lines (······) represent no added nitrate incubations, and the solid lines (_________) represent the system with 20 mM nitrate added. The errors shown represent the standard deviation on the triplicate measurements.

**Figure 3:**
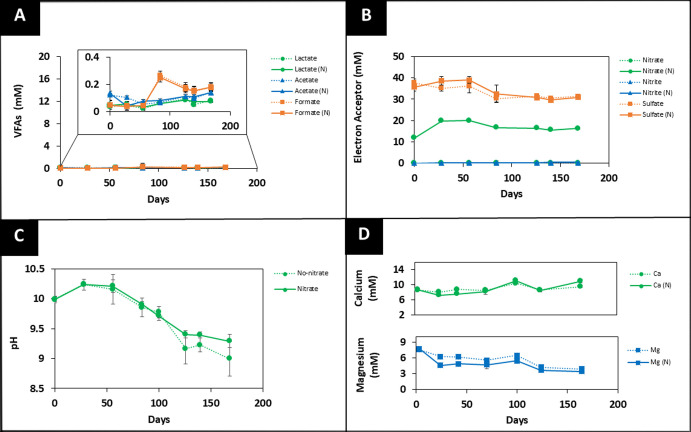
Geochemical data for the no-carbon system. (A) VFA, (B) electron acceptor, (C) pH, and (D) Ca^2+^ and Mg^2+^ concentrations from a 6 month incubated system at 25 °C are presented. (“N” is used to represent the system with added nitrate). The dotted lines (······) represent no added nitrate incubations, and the solid lines (______) represent systems with 20 mM nitrate added. The errors shown represent the standard deviation on the triplicate measurement.

ICP-AES analysis confirmed an increase in Ca^2+^ ion concentrations from 8.7 ± 0.4 mM to 15.7 ± 0.9 mM in the low-carbon system (Figure 2D), although no significant structural changes in the low-carbon cement tablets were detected through 2D and 3D imaging, as discussed in Section 3.2. The Ca^2+^ ion concentration in the no-carbon system remained constant at approximately 9.1 ± 0.6 mM, and Mg^2+^ ion levels also remained constant after the initial removal from groundwater for the first month (Figure 3D).

Nitrate reduction was absent in the no-added electron acceptor controls (Figures 1B, 2B, and 3B; dotted line). In all controls, a gradual increase in formate (Figures 1A, 2A, 3A; dotted line) and a decrease in pH (Figures 1C, 2C, 3C; dotted line) were observed, indicating minor microbial processes were active. It is worth noting that none of the controls were sterile (as the cement tablets were not sterilized before use), 5 g of sediment inoculum was present in all bottles, and IC analysis of the 5 g of sediment inoculum present in all the bottles contained 0.04 mM formate and other VFAs (Supplementary Table S2). This suggests trace electron donors were present in the indigenous sediment, and this does not capture other potential carbon sources that are not detectable by IC, e.g., alcohols. Overall, the small changes in the controls were not significant when compared to the microbially active systems with high carbon and nitrate concentrations. These changes were accompanied by an increase in Mg^2+^ and Ca^2+^ ion concentration (Figures 1D, 2D, and 3D; dotted line).

### 3.2 Cement Microstructure Characterization

Solid-phase analyses of the cement microstructure were conducted to complement the geochemical data of solution obtained from the microcosm incubations. A visual inspection of the high-carbon system revealed extensive pale deposits on the cement surface (Figure 4A). SEM images showed the morphology of the newly formed minerals (Figure 4B), which were rhombohedral in shape and calcite-like, as reported in other high pH biomineralization studies.^46,61,62^ EDS analysis of these particles suggested that the minerals were rich in calcium and magnesium, consistent with carbonate deposits, e.g., Mg and Ca carbonates (Figure 4C). To further investigate the distribution of these elements across the surface of cement samples, thin sections were prepared and analyzed using SEM-EDS mapping. The elemental mapping of the cross-section revealed that calcium (Figure 4E) and, notably, magnesium (Figure 4F) were present on the outer rim of the cement tablet. Mg in the rim was clearly concentrated compared to the bulk sample. Additionally, while a Ca rim is clearly visible (Figure 4E), and accompanied by an enhanced Ca EDS signal (Figure 4C, vs Figures 5C and 6C), it is noted that the bulk of the cement is also rich in Ca, attributing a more subtle appearance compared with the Mg (Figure 4F). Further analysis using FTIR identified calcite and confirmed the presence of calcium carbonate formations on the cement surface (Supplementary Figure S5).

**Figure 4:**
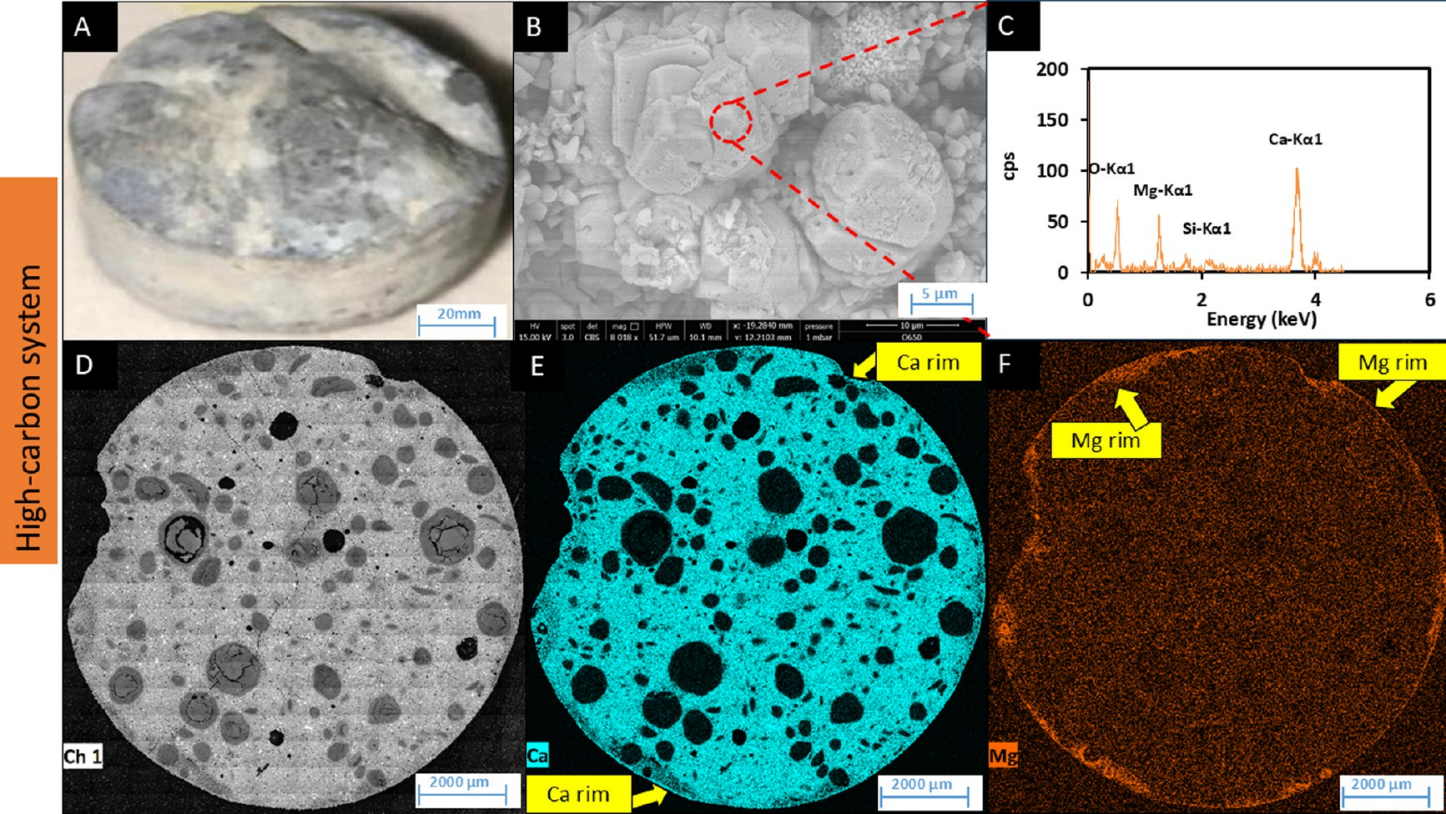
SEM and EDS analyses of cement tablets from the high-carbon system. (A) Cement tablet surface after 6 months incubation, (B) SEM-BSE image of new mineral on the surface, (C) EDS spectra for semiquantitative analysis of new mineral deposited, (D) EDS-map showing Ca distribution, (E) EDS-map showing Ca distribution, and (F) EDS-map showing Mg distribution.

**Figure 5:**
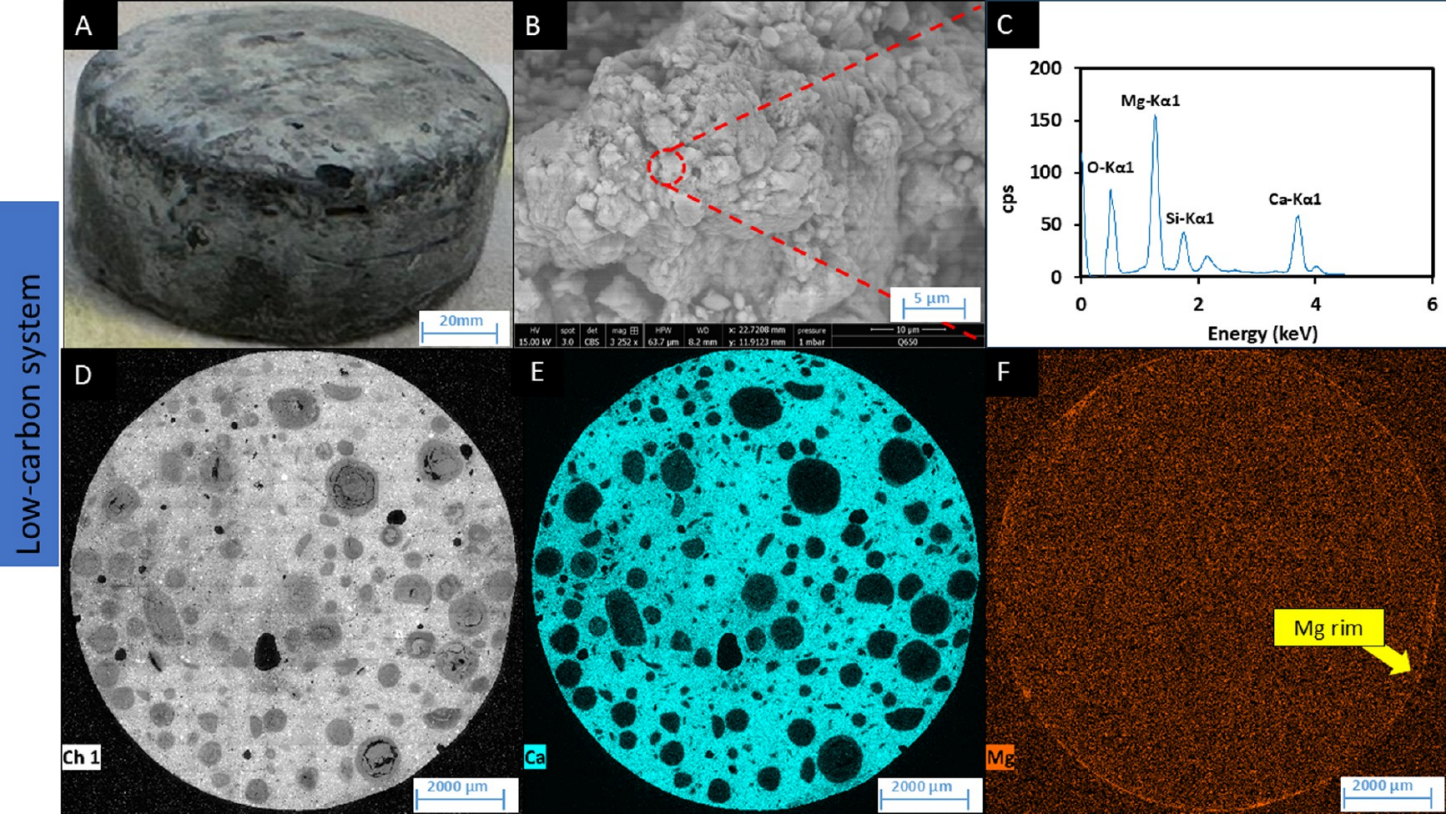
SEM and EDS analyses of cement tablets from the low-carbon system. (A) Cement tablet surface after 6 months incubation, (B) SEM-BSE image of new mineral on the surface, (C) EDS spectra for semiquantitative analysis of new mineral deposited, (D) EDS-map of the cross-section of the cement tablets, (E) EDS-map showing Ca distribution, and (F) EDS-map showing Mg distribution.

**Figure 6:**
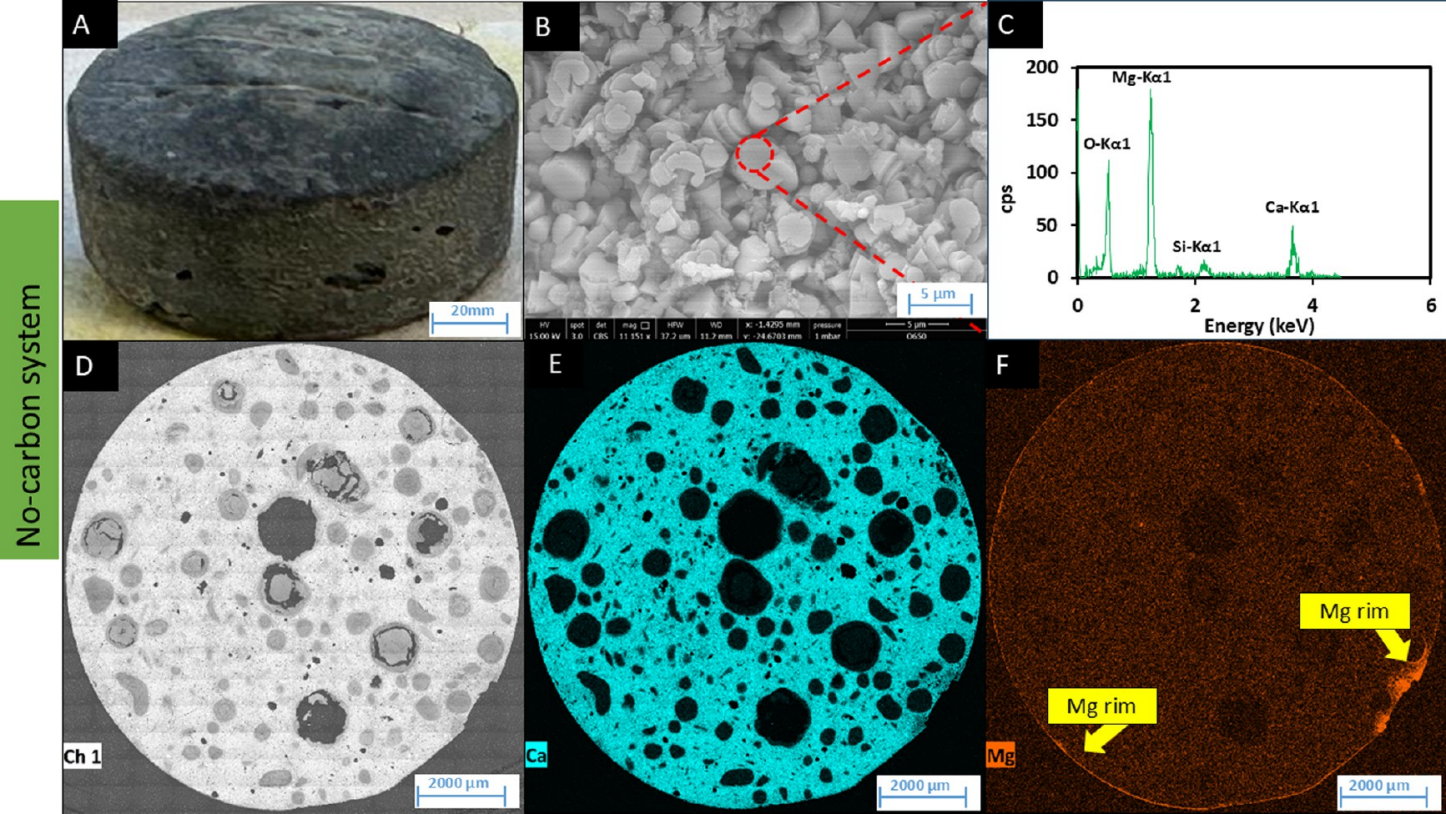
SEM and EDS analyses of cement tablets from the no-carbon system. (A) Cement tablet surface after 6 months incubation, (B) SEM-BSE image of new mineral on the surface, (C) EDS spectra for semiquantitative analysis of new mineral deposited, (D) EDS-map of the cross-section of the cement tablets, (E) EDS-map showing Ca distribution, and (F) EDS-map showing Mg distribution.

Visual inspection of the low-carbon and no-carbon systems revealed a much thinner and uneven layer of pale precipitate on the cement surface (Figures 5A and 6A). SEM-EDS analysis indicated that the precipitate was rich in magnesium and calcium (Figures 5C and 6C), while EDS mapping confirmed an enhanced, thin boundary layer of magnesium (Figures 5F and 6F); however, no Ca enrichment was detected (Figures 5E and 6E). Further characterization of the precipitate was challenging, given the small amounts of biomineral precipitated. Similarly, a low level of a pale mineral deposit was observed in all no-nitrate control systems, and further analyses were not possible.

### 3.3 2D and 3D Imaging

Geochemical and mineralogical data indicated that new minerals precipitated in all of the systems. This was further confirmed by SEM imaging. Images of the day 0 cement tablets showed a smooth surface (Figure 7A), whereas, after six months of incubation, the surface texture of all systems had become rough. This was presumably due to alteration and Ca- and Mg-carbonate mineralization (Figure 7B–D), which is supported by geochemical data that showed Ca^2+^ and Mg^2+^ had precipitated over time. A detailed inspection of crack healing revealed that Ca- and Mg-carbonate precipitates sealed cracks present at the start of the experiment in the high-carbon system over time (Figure 7F). However, this healing process was not observed in the low- and no-carbon systems (Figure 7G,H). Crack healing can be advantageous for maintaining the integrity of the cement structure for a longer period.^63^

**Figure 7:**
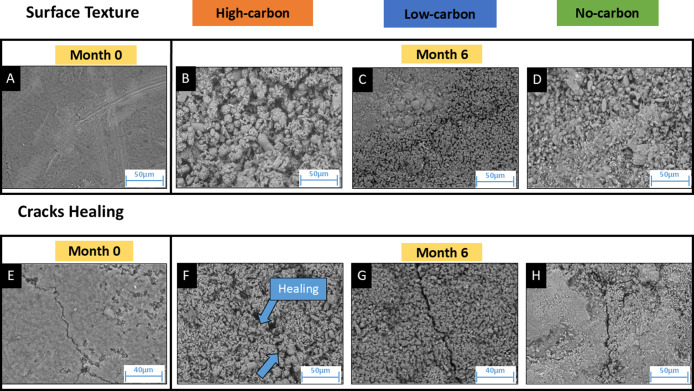
Surface texture (top panels) and crack healing (bottom panels) of tablet cement surfaces imaged at 6 months using SEM under different incubation regimes. (A) rpresents the surface of month 0 of the cement tablet. After 6 months, (B) a high-carbon system, (C) a low-carbon system, and (D) a no-carbon system surface texture is shown. (E) represents the surface of month 0 with a crack in the cement tablet. After 6 months, (F) high-carbon system, (G) low-carbon system, and (H) no-carbon system crack-healing processes are compared.

To further explore surface mineral deposition, samples were analyzed by 3D μ-XCT. 3D data of cement tablets (6 mm in height and 10 mm in diameter) from the high- and .low-carbon systems were segmented to examine the extent of surface deposits and quantify pore size distribution. The orthoslice image of the cement tablet shows the distribution of the different unaltered cement phases (Figure 8A). Segmentation revealed that surface deposits of carbonate were more extensive in tablets from the high-carbon system (Figure 8B) than those from the low-carbon system (Figure 8C) after six months of exposure. Additionally, an analysis of pore size diameter distribution indicated that the high-carbon system showed a decrease in the number of pores with larger diameters, between 200 and 400 μm, after six months compared to the one-month sample (Figure 9A,B). This suggested that pore filling occurred over time in the high-carbon system. This was further supported by the increased carbonate deposition observed on the cement surface over six months (Figure 8B) and geochemical data showing a decrease in Ca^2+^ ion concentration in solution (Figure 1D). In contrast, the low-carbon system showed no substantial change in the number of pores sized 200 and 400 μm in diameter between the one- and six-month samples (Figure 9C,D).

**Figure 8:**
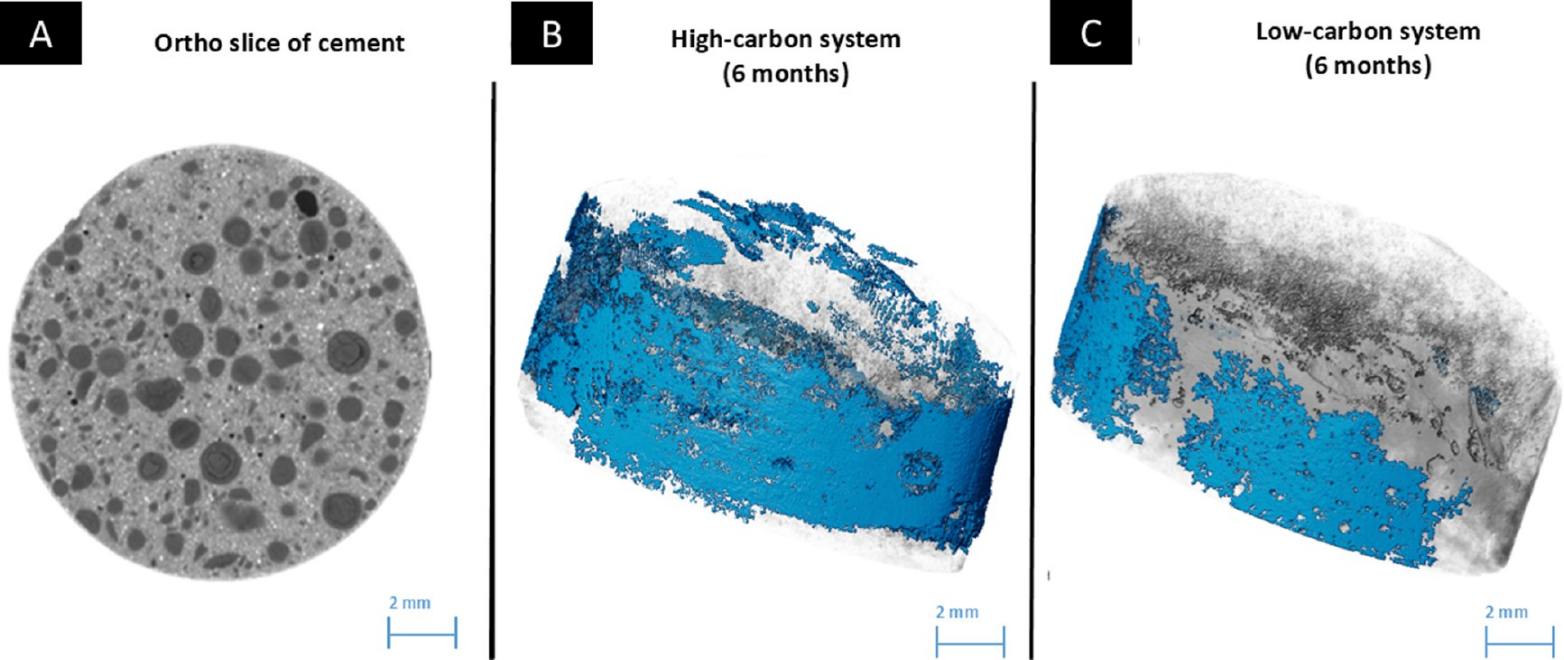
XCT scanning of tablets for 3D imaging. (A) Ortho slice of a volume section of an unaltered cement tablet. The different gray scales represent different phases present in the sample. Segmentation of the carbonate phase (artificially colored blue) deposited in six months on the surface of (B) High-carbon system and (C) Low-carbon systems.

**Figure 9:**
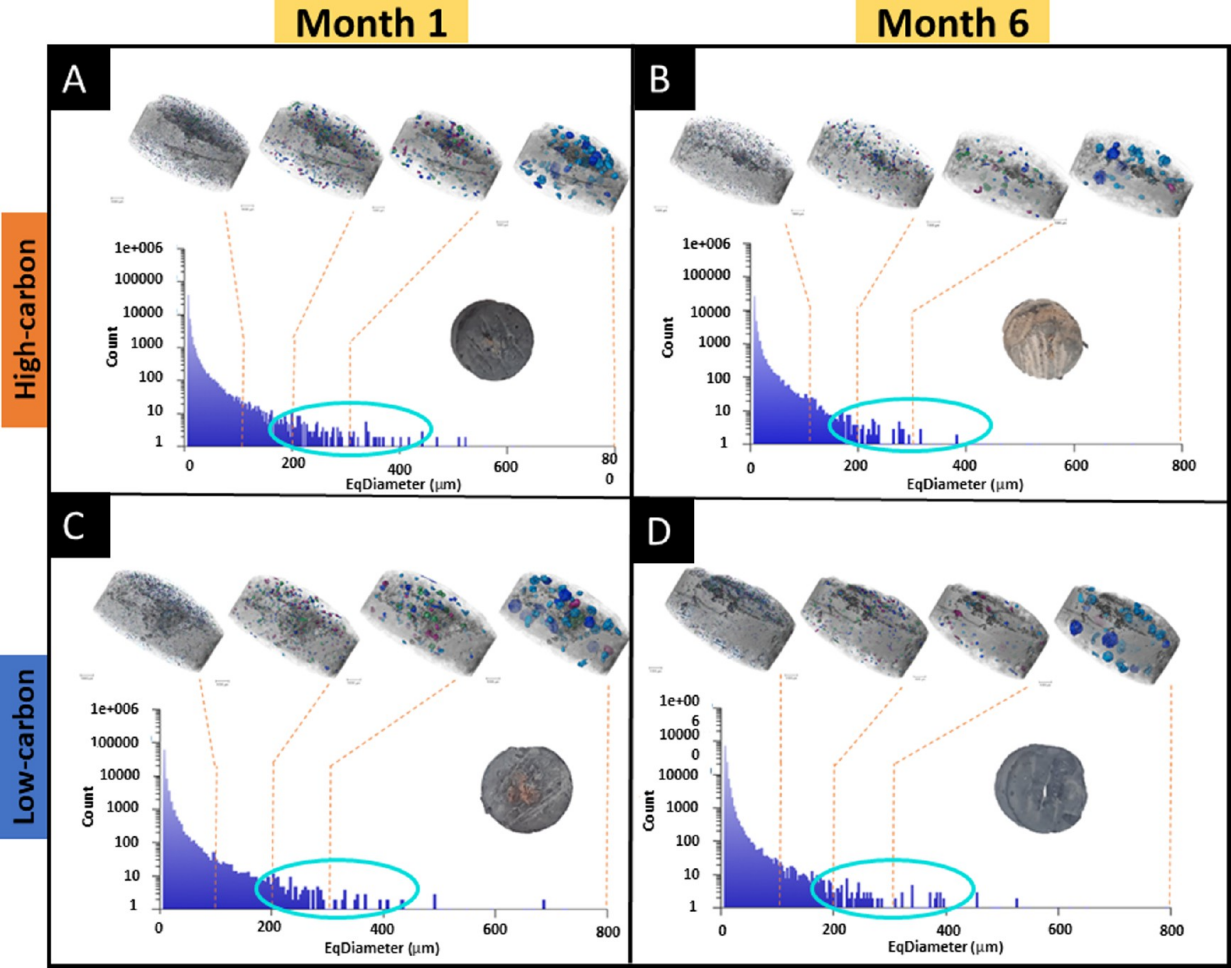
Comparison of one- and six-month 3D XCT images of segmented pore spaces (0–100, 100–200, 200–300, and 300–800 μm EqDiameter measurements). Semilog graph of pore diameter v/s counts for (A,B) high-carbon and (C,D) low-carbon system, with added nitrate. The blue circle over EqDiameter from 200 to 400 μm covers the region to show obvious differences in pore count values.

The comparison focused on pore sizes between 200 and 400 μm diameter, as in all samples, smaller pores (<200 μm) were too densely distributed to allow a clear comparison, and larger pores (>400 μm) were deemed less relevant due to their size relative to the cement tablet diameter (1 cm). Visual inspection of segmented 3D images also confirmed a reduction in pore count near the outer boundary of the high-carbon system compared to the low-carbon system, which was further confirmed by the graphical plot of the number of pores present in different ranges (Supplementary Figure S6). This indicates that surface carbonation reduced the pore count near the boundary. However, it is worth noting that over time, as carbonate minerals precipitate, the rate of further carbonation may be reduced due to increasing cement impermeability, which may prevent the influx of fresh carbonate/carbon and microbial activity at the cement surface. Crack healing and pore filling are potentially important considerations for GDFs. Healed cracks are expected to limit groundwater infiltration, thereby reducing radionuclide mobility. However, sealed pores and healed cracks may also hinder gas migration (e.g., hydrogen, nitrogen, and methane), potentially leading to increased internal pressure.

### 3.4 Microbial Community and 16S rRNA Gene Sequencing

PCR-based high-throughput 16S rRNA gene sequencing was used to characterize the microbial communities initially present in the high pH sediment inoculum (experimental start point) versus community changes in samples obtained on days 114 and 164 (experimental midpoint and end-points). The predominant phylogenetic classes identified in the initial inoculum comprised Bacteroidia (50–60%) and Gammaproteobacteria (20–30%) (Figure 10).

**Figure 10:**
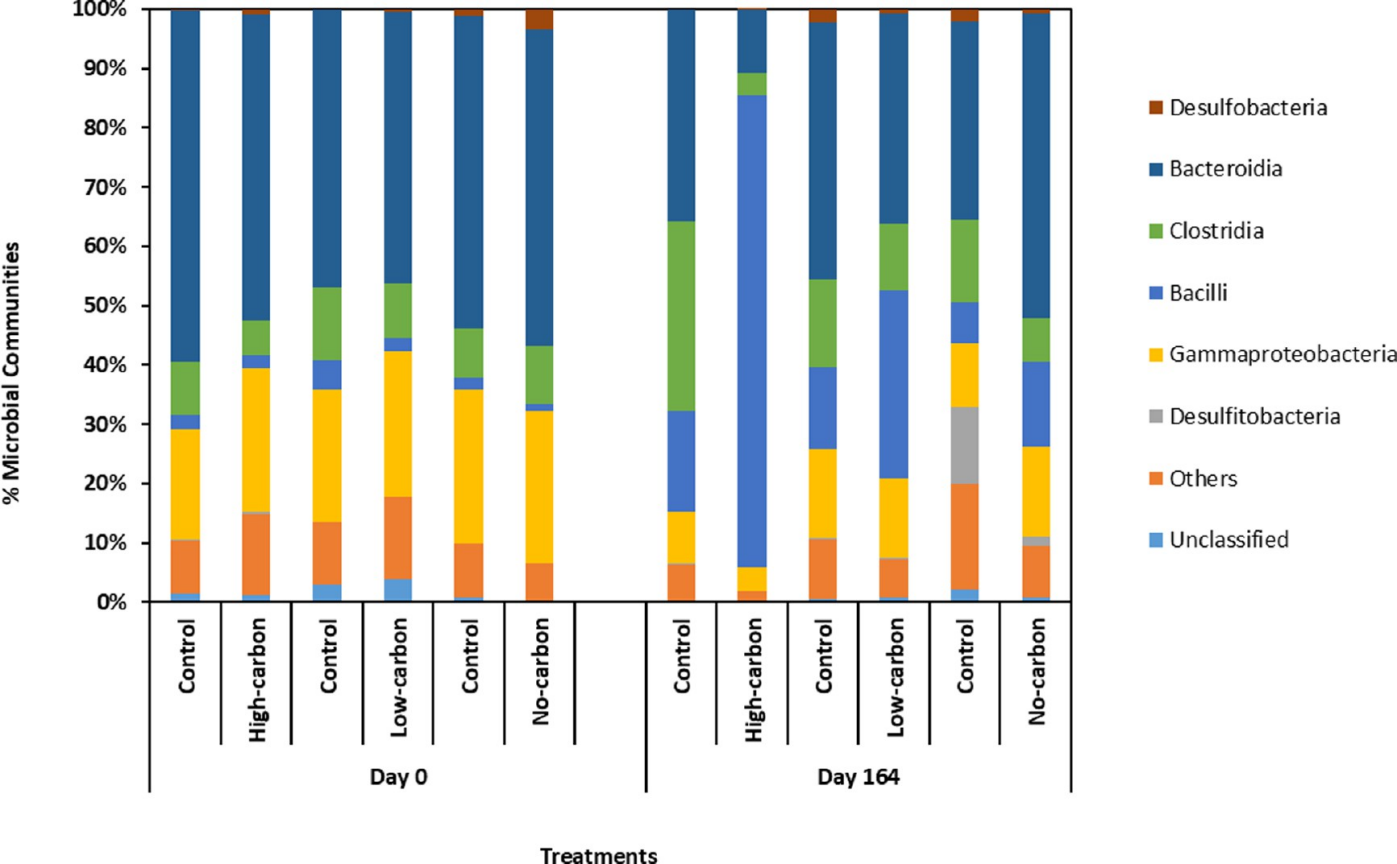
Microcosm microbial community profiles from analysis of 16S rRNA genes amplified by PCR from the incubation systems at day 0 and day 164 (class level affiliations).

After six months, the high-carbon system was dominated by Bacilli (87%), followed by Bacteroidia (8%). At the genus level, sequences closely matching members of the genus *Anaerobacillus* (99.6%) dominated (Supplementary Figure S7). At the species level, there was a close sequence match to *Anaerobacillus isosaccharinicus* (99% identity match over 253 bases), a known alkaliphile capable of utilizing lactate as an electron donor and reducing nitrate as an electron acceptor to nitrite.^64,65^ Many species within the *Anaerobacillus* genus are alkaliphilic heterotrophic nitrate reducers. The no-nitrate control of the high-carbon system was rich in Clostridia (35%) and Bacteroidia (33%) (Figure 10), consistent with the decrease in lactate, likely by fermentation reactions.^66^

In the low-carbon system, subtle shifts in the community were observed: Bacteroidia (37%) dominated after six months, followed by Bacilli (30%) (Figure 10). The sequences relating to Bacteroidia could not be resolved at the genus level. For the Bacilli, again, the sequences most closely matched the genus *Anaerobacillus*.^64,65^ However, in this system, while geochemical data indicated microbial activity was limited (Figure 5), it is noted that low levels of activity may have been stimulated by H_2_ supplied as an electron donor (with nitrate), reflecting the observed shift in the microbial ecology for this system (Figure 10). The no-nitrate and no-added carbon controls did not show significant changes in the major microbial community at the end-point compared to the starting point, confirming that electron donor/acceptor amendments were driving changes in the microbial community in the microbially stimulated experiments. Notably, a small percentage of organisms affiliated with *Desulfitobacteriia* were present in the low-carbon system. *Desulfitobacterium* spp. show a highly versatile energy metabolism with the capacity to use different electron donors and acceptors present in the Harpur Hill sediment and can even grow fermentatively.^67^ Overall, this suggests that the high-carbon system had a higher abundance of *Bacilli*, which are well-known for their role in MICP, compared to the low-carbon and no-carbon systems.

## 4 Conclusion

This study provides new insights into how microbial metabolism behaves in low-pH cement systems under geological repository-relevant conditions, particularly in relation to carbon and electron acceptor availability. In microcosms buffered at pH 10.5–11 and containing low-pH CEBAMA mix cement pellets, alkaliphilic microbes were readily stimulated by the addition of a model organic carbon source and nitrate as electron acceptor, leading to MICP. The extent of MICP followed the trend: high-carbon system > low-carbon system > no-carbon system. This confirms that microbial activity and associated carbonate biomineralization are strongly dependent on the availability of organic carbon and the presence of an electron acceptor. In contrast, when these substrates were absent, microbial metabolism was limited, resulting in negligible CO_2_ production and no observable carbonate biomineralization, crack healing, or pore filling.

This study showed that an adequate organic carbon source is important for supporting cement crack-healing processes during this study period. In the low-carbon system, even high-energy-yielding electron donors such as hydrogen—relevant to GDFs via steel cannister corrosion—did not significantly support microbial activity over the experimental time scales required to promote MICP. However, when supplemented with higher levels of organic carbon—for example, through localized pockets of organics within the heterogeneous waste—anaerobes, including nitrate-reducing bacteria (as demonstrated in this study), were able to promote crack healing and pore filling. This is likely beneficial in a GDF, as it may reduce contaminant migration pathways and help in maintaining the structural integrity of the waste form or repository. However, the extent to which MICP may occur in a dynamic repository system, where substrates for microbial metabolism can be replenished via a groundwater flow, remains unclear. On this note, nitrate, the MICP supporting electron acceptor utilized in this study, can be present in some ILW wastes.^68^ This could be supplemented by sulfate from the waste inventory/cement or groundwater ingress, but in this study, sulfate reduction did not seem to sustain microbial activity over the time frame studied. Finally, extensive MICP, which reduces overall pore volume, may also have wider impacts on processes such as gas diffusion, and further research is needed to fully understand potential implications. Despite these uncertainties, the present study suggests that microbial activity does not pose a significant threat to cement integrity over the six month period of our experiments, even under conditions accelerated by the addition of elevated labile organic carbon (as an electron donor) and nitrate (as an electron acceptor). On the contrary, microbial metabolism under these conditions leads to microbial-induced carbonate precipitation (MICP), which could be advantageous for repository stability.

## Supplementary Material



## Notes

XRD on CEBAMA reference mix and its components, microcosm summary and Harpur hill sediments analysis, μ-XCT protocol, PHREEQC data for the experiment end-point, FTIR analysis of carbonate deposition on cement tablets, pore count analysis, microbial community data (genus level), controls for a 6 month experiment with autoclaved Harpur Hill sediment, and controls for a 6 month experiment to monitor cement leaching (PDF)

## References

[1] Waste-Report-010223 2022. https://ukinventory.nda.gov.uk/wp-content/uploads/2023/02/2022-Waste-Report-010223.pdf (accessed 2024–12–24).

[2] Main-UKRWI-Report_Final­(2020).Pdf. https://ukinventory.nda.gov.uk/wp-content/uploads/2021/03/20201222-Official-Rep-PO023346-Main-UKRWI-Report_Final.pdf (accessed Dec,24 2024).

[3] Status and Trends in Spent Fuel and Radioactive Waste Management.

[4] Changes since 2019. UK Radioactive Waste & Materials Inventory. https://ukinventory.nda.gov.uk/data-hub/changes-since-2019/ (accessed Dec,24 2024).

[5] Scientific and Technical Basis for the near Surface Disposal of Low and Intermediate Level Waste; Internationale Atomenergie-Organisation, Internationale Atomenergie-Organisation, Eds.; Technical reports series/International Atomic Energy Agency; International Atomic Energy Agency: Vienna, 2002.

[6] Ojovan M. I., Steinmetz H. J. (2022). Approaches
to Disposal of Nuclear Waste. Energies.

[7] Kozak, M. W. 16 - Safety Assessment for near-Surface Disposal of Low and Intermediate Level Wastes. In Geological Repository Systems for Safe Disposal of Spent Nuclear Fuels and Radioactive Waste, Second ed. ed.; Apted, M. J. , Ahn, J. , Eds.; Woodhead Publishing Series in Energy; Woodhead Publishing, 2017; pp 475–498.

[8] Glasser F. P., Atkins M. (1994). Cements in Radioactive Waste Disposal. MRS Bull..

[9] Ma B., Provis J. L., Wang D., Kosakowski G. (2024). The Essential
Role of Cement-Based Materials in a Radioactive Waste Repository. Npj Mater. Sustain..

[10] Marsh A. I., Williams L. G., Lawrence J. A. (2021). The Important Role
and Performance
of Engineered Barriers in a UK Geological Disposal Facility for Higher
Activity Radioactive Waste. Prog. Nucl. Energy.

[11] Grauer R., Knecht B., Kreis P., Simpson J. P. (1990). Hydrogen
Evolution
From Corrosion of Iron and Steel in Intermediate Level Waste Repositories. MRS Online Proc. Libr. OPL.

[12] Xu D., Gu T., Lovley D. R. (2023). Microbially
Mediated Metal Corrosion. Nat. Rev. Microbiol..

[13] Maes M., De Belie N. (2014). Resistance of Concrete
and Mortar against Combined
Attack of Chloride and Sodium Sulphate. Cem.
Concr. Compos..

[14] Stanaszek-Tomal E., Fiertak M. (2016). Biological Corrosion
in the Sewage System and the Sewage
Treatment Plant. Procedia Eng..

[15] Madsen F. T. (1998). Clay Mineralogical
Investigations Related to Nuclear Waste Disposal. Clay Miner..

[16] Savage, D. ; Benbow, S. Low pH Cements; Swedish Nuclear Power Inspectorate; Sweden, 2007, 1104–1374, p 55.

[17] Vehmas T., Montoya V., Alonso M. C., Vašíček R., Rastrick E., Gaboreau S., Večerník P., Leivo M., Holt E., Fink N., Ait Mouheb N., Svoboda J., Read D., Červinka R., Vasconcelos R., Corkhill C. (2020). Characterization of Cebama Low-pH
Reference Concrete and Assessment of Its Alteration with Representative
Waters in Radioactive Waste Repositories. Appl.
Geochem..

[18] Vasconcelos R. G. W., Walkley B., Day S., Tang C. C., Paraskevoulakos H., Gardner L. J., Corkhill C. L. (2020). 18-Month
Hydration of a Low-pH Cement
for Geological Disposal of Radioactive Waste: The Cebama Reference
Cement. Appl. Geochem..

[19] Onofrei M., Gray M. N., Coons W. E., Alcorn S. R. (1992). High Performance
Cement-Based Grouts for Use in a Nuclear Waste Disposal Facility. Waste Manag.

[20] Cau
Dit Coumes C., Courtois S., Nectoux D., Leclercq S., Bourbon X. (2006). Formulating a Low-Alkalinity, High-Resistance and Low-Heat
Concrete for Radioactive Waste Repositories. Cem. Concr. Res..

[21] Othman H., Sabrah T., Marzouk H. (2019). Conceptual
Design of Ultra-High Performance
Fiber Reinforced Concrete Nuclear Waste Container. Nucl. Eng. Technol..

[22] Liu Z., Shao J., Zha W., Xie S., Bourbon X., Camps G. (2021). Shear Strength of Interface between High-Performance Concrete and
Claystone in the Context of a French Radioactive Waste Repository
Project. Géotechnique.

[23] Larbi J. A., Fraay A. L. A., Bijen J. M. J. M. (1990). The Chemistry
of the Pore Fluid of
Silica Fume-Blended Cement Systems. Cem. Concr.
Res..

[24] Yajun J. (2004). Simulation
of Silica Fume Blended Cement Hydration. Mater.
Struct..

[25] Pellegrini, S. . Corrosion Behavior of Carbon Steel Rebar in Calcium Sulfoaluminate CSA-Based Systems, University of Bergamo, 2017.

[26] Akerele D.
D., Aguayo F. (2024). Evaluating
the Impact of CO2 on Calcium SulphoAluminate
(CSA) Concrete. Buildings.

[27] Khan M. S. H., Nguyen Q. D., Castel A. (2020). Performance of Limestone
Calcined
Clay Blended Cement-Based Concrete against Carbonation. Adv. Cem. Res..

[28] El
Bitouri Y., Buffo-Lacarrière L., Sellier A., Bourbon X. (2016). Modelling of Chemo-Mechanical Behaviour of Low pH Concretes. Cem. Concr. Res..

[29] Vasconcelos, R. ; Walkley, B. ; Hyatt, N. ; Provis, J. ; Corkhill, C. The Physico-Chemical Evolution of a Low-pH Cement in Contact with Groundwater. Deliverable nD4. 13 Draft of the 3rd Annual Project Workshop Proceeding 2018, 71.

[30] Duro L., Altmaier M., Holt E., Mäder U., Claret F., Grambow B., Idiart A., Valls A., Montoya V. (2020). Contribution of the Results of the CEBAMA Project to
Decrease Uncertainties in the Safety Case and Performance Assessment
of Radioactive Waste Repositories. Appl. Geochem..

[31] Ichikawa N., Hamamoto T. (2021). Safety Function of
Cementitious Materials and the Analytical
Assessment of Long-Term Evolution of Cement-Bentonite Interface for
Geological Disposal in Japan. J. Adv. Concr.
Technol..

[32] Rath S., Sancharoen P., Klomjit P., Tangtermsirikul S. (2021). Effects of
Carbonation on Corrosion Rate of Reinforcing Steel in Different Concrete
and Repair Materials. Eng. J..

[33] Rothschild L. J., Mancinelli R. L. (2001). Life in
Extreme Environments. Nature.

[34] Rizoulis A., Steele H. M., Morris K., Lloyd J. R. (2012). The Potential Impact
of Anaerobic Microbial Metabolism during the Geological Disposal of
Intermediate-Level Waste. Mineral. Mag..

[35] Lloyd, J. R. ; Cherkouk, A. The Microbiology of Nuclear Waste Disposal; Elsevier, 2020.

[36] Butterworth S. J., Barton F., Lloyd J. R. (2023). Extremophilic Microbial Metabolism
and Radioactive Waste Disposal. Extremophiles.

[37] Davis J. L., Nica D., Shields K., Roberts D. J. (1998). Analysis of Concrete
from Corroded Sewer Pipe. Int. Biodeterior.
Biodegrad..

[38] Glasser F. P., Marchand J., Samson E. (2008). Durability
of Concrete — Degradation
Phenomena Involving Detrimental Chemical Reactions. Cem. Concr. Res..

[39] Turick C. E., Berry C. J. (2016). Review of Concrete
Biodeterioration in Relation to
Nuclear Waste. J. Environ. Radioact..

[40] Jonkers, H. M. Self Healing Concrete: A Biological Approach. In Self Healing Materials: An Alternative Approach to 20 Centuries of Materials Science; van der Zwaag, S. , Ed.; Springer Netherlands: Dordrecht, 2007; pp 195–204.

[41] De
Muynck W., De Belie N., Verstraete W. (2010). Microbial
Carbonate Precipitation in Construction Materials: A Review. Ecol. Eng..

[42] Dhami N. K., Reddy M. S., Mukherjee A. (2013). Biomineralization
of Calcium Carbonate
Polymorphs by the Bacterial Strains Isolated from Calcareous Sites. J. Microbiol. Biotechnol..

[43] Seifan M., Berenjian A. (2018). Application of Microbially Induced Calcium Carbonate
Precipitation in Designing Bio Self-Healing Concrete. World J. Microbiol. Biotechnol..

[44] Zhang Y. S., Liu Y., Sun X. D., Zeng W., Xing H. P., Lin J. Z., Kang S. B., Yu L. (2024). Application
of Microbially Induced
Calcium Carbonate Precipitation (MICP) Technique in Concrete Crack
Repair: A Review. Constr. Build. Mater..

[45] Alonso M. J. C., Ortiz C. E. L., Perez S. O. G., Narayanasamy R., Fajardo San Miguel G.
D. J., Hernández H. H., Balagurusamy N. (2018). Improved Strength and Durability of Concrete through
Metabolic Activity of Ureolytic Bacteria. Environ.
Sci. Pollut. Res..

[46] Nasser A. A., Sorour N. M., Saafan M. A., Abbas R. N. (2022). Microbially-Induced-Calcite-Precipitation
(MICP): A Biotechnological Approach to Enhance the Durability of Concrete
Using Bacillus Pasteurii and Bacillus Sphaericus. Heliyon.

[47] Bassil N. M., Bryan N., Lloyd J. R. (2015). Microbial
Degradation of Isosaccharinic
Acid at High pH. ISME J..

[48] Small J. S., Nykyri M., Vikman M., Itävaara M., Heikinheimo L. (2017). The Biogeochemistry of Gas Generation from Low-Level
Nuclear Waste: Modelling after 18 Years Study under in Situ Conditions. Appl. Geochem..

[49] Durban N., Rafrafi Y., Rizoulis A., Albrecht A., Robinet J.-C., Lloyd J. R., Bertron A., Erable B. (2018). Nitrate and Nitrite
Reduction at High pH in a Cementitious Environment by a Microbial
Microcosm. Int. Biodeterior. Biodegrad..

[50] Byrd N., Lloyd J. R., Small J. S., Taylor F., Bagshaw H., Boothman C., Morris K. (2021). Microbial
Degradation of Citric Acid
in Low Level Radioactive Waste Disposal: Impact on Biomineralization
Reactions. Front. Microbiol..

[51] Durban N., Bertron A., Sonois-Mazars V., Schiettekatte M., Matar G., Albina P., Albrecht A., Robinet J.-C., Erable B. (2023). Biogeochemical Interactions between Aged Cementitious
Materials and Sulfate Reducing Microbial Community with Propionate
as Electron Donor in the Context of Nuclear Waste Repository. Appl. Geochem..

[52] Assessment, U. E. N. C. for E . Description of input and examples for PHREEQC version 3: a computer program for speciation, batch-reaction, one-dimensional transport, and inverse geochemical calculations, 2013. https://hero.epa.gov/hero/index.cfm/reference/details/reference_id/7676153 (accessed 2024–12–23).

[53] Giffaut E., Grivé M., Blanc Ph., Vieillard Ph., Colàs E., Gailhanou H., Gaboreau S., Marty N., Madé B., Duro L. (2014). Andra Thermodynamic Database for
Performance Assessment: ThermoChimie. Appl.
Geochem..

[54] Guide to Reference Groundwater and Porewater Compositions in Support of the UK GDF Programme Geo-Disposal Radwaste Programme; UK Research and Innovation, 2022.

[55] Williamson A. J., Lloyd J. R., Boothman C., Law G. T. W., Shaw S., Small J. S., Vettese G. F., Williams H. A., Morris K. (2021). Biogeochemical
Cycling of 99Tc in Alkaline Sediments. Environ.
Sci. Technol..

[56] Lane, D. J. 16S/23S rRNA Sequencing, Nucleic Acid Techniques in Bacterial Systematic; John Wiley and Sons, Eds., Stackebrandt, E. ; Goodfellow, M. , Eds., 1991; pp 115–174.

[57] Caporaso J. G., Lauber C. L., Walters W. A., Berg-Lyons D., Lozupone C. A., Turnbaugh P. J., Fierer N., Knight R. (2011). Global Patterns
of 16S rRNA Diversity at a Depth of Millions of Sequences per Sample. Proc. Natl. Acad. Sci. U.S.A..

[58] Caporaso J. G., Lauber C. L., Walters W. A., Berg-Lyons D., Huntley J., Fierer N., Owens S. M., Betley J., Fraser L., Bauer M., Gormley N., Gilbert J. A., Smith G., Knight R. (2012). Ultra-High-Throughput
Microbial Community
Analysis on the Illumina HiSeq and MiSeq Platforms. ISME J..

[59] Kozich J. J., Westcott S. L., Baxter N. T., Highlander S. K., Schloss P. D. (2013). Development of a Dual-Index Sequencing Strategy and
Curation Pipeline for Analyzing Amplicon Sequence Data on the MiSeq
Illumina Sequencing Platform. Appl. Environ.
Microbiol..

[60] Akunna J. C., Bizeau C., Moletta R. (1993). Nitrate and Nitrite Reductions with
Anaerobic Sludge Using Various Carbon Sources: Glucose, Glycerol,
Acetic Acid, Lactic Acid and Methanol. Water
Res..

[61] Rodriguez-Navarro C., Jimenez-Lopez C., Rodriguez-Navarro A., Gonzalez-Muñoz M. T., Rodriguez-Gallego M. (2007). Bacterially
Mediated Mineralization of Vaterite. Geochim.
Cosmochim. Acta.

[62] Sohail M. G., Disi Z. A., Zouari N., Nuaimi N. A., Kahraman R., Gencturk B., Rodrigues D. F., Yildirim Y. (2022). Bio Self-Healing Concrete
Using MICP by an Indigenous Bacillus Cereus Strain Isolated from Qatari
Soil. Constr. Build. Mater..

[63] Gjørv, O. E. ; Sakai, K. ; Banthia, N. Concrete Under Severe Conditions 2: Environment and Loading: Proceedings of the Second International Conference on Concrete Under Severe Conditions, CONSEC ’98, Tromsø, Norway, June 21–24, 1998; CRC Press, 1998.

[64] Zavarzina D. G., Tourova T. P., Kolganova T. V., Boulygina E. S., Zhilina T. N. (2009). Description of Anaerobacillus Alkalilacustre
Gen. Nov.,
Sp. Nov.—Strictly Anaerobic Diazotrophic Bacillus Isolated
from Soda Lake and Transfer of Bacillus Arseniciselenatis, Bacillus
Macyae, and Bacillus Alkalidiazotrophicus to Anaerobacillus as the
New Combinations A. Arseniciselenatis Comb. Nov., A. Macyae Comb.
Nov., and A. Alkalidiazotrophicus Comb. Nov. Microbiology.

[65] Bassil N. M., Lloyd J. R. (2019). Anaerobacillus Isosaccharinicus
Sp. Nov., an Alkaliphilic
Bacterium Which Degrades Isosaccharinic Acid. Int. J. Syst. Evol. Microbiol..

[66] Mei N., Postec A., Erauso G., Joseph M., Pelletier B., Payri C., Ollivier B., Quéméneur M. (2016). Serpentinicella
Alkaliphila Gen. Nov., Sp. Nov., a Novel Alkaliphilic Anaerobic Bacterium
Isolated from the Serpentinite-Hosted Prony Hydrothermal Field, New
Caledonia. Int. J. Syst. Evol. Microbiol..

[67] Willemin M. S., Hamelin R., Armand F., Holliger C., Maillard J. (2023). Proteome Adaptations
of the Organohalide-Respiring Desulfitobacterium Hafniense Strain
DCB-2 to Various Energy Metabolisms. Front.
Microbiol..

[68] Bertron A., Jacquemet N., Erable B., Sablayrolles C., Escadeillas G., Albrecht A. (2014). Reactivity of Nitrate and Organic
Acids at the Concrete–Bitumen Interface of a Nuclear Waste
Repository Cell. Nucl. Eng. Des..

