# Supplementary material for: Microbially-Induced Carbonate Precipitation in Low pH Cement; Potential for Self-Healing in Radioactive Waste Geodisposal Systems
Source: ACS Omega. 2025 Nov 19;10(47):57011–24. doi: 10.1021/acsomega.5c04312 (PMC12676360; doi:10.1021/acsomega.5c04312)
Supplement: Supplementary file 1 [file ao5c04312_si_001.pdf]

Microbial-induced carbonate precipitation in low pH cement; potential for self-healing in radioactive waste geodisposal systems

Ananya Singh [1], Natalie Byrd [1], Dirk Engelberg [2], Christopher Boothman [3], Samuel Shaw [1], Katherine Morris[1], Jonathan R. Lloyd [1*]*

1. Department of Earth and Environmental Science, Radioactive Waste Disposal and Environmental Remediation (RADER) National Nuclear User Facility and Williamson Research Centre, The University of Manchester, Manchester M13 9PL, UK
2. Metallurgy and Corrosion, Department of Materials, The University of Manchester, Manchester M13 9PL, UK
3. Manchester Institute of Biotechnology, The University of Manchester, Manchester M13 9PL, UK

Supplementary Material

1. XRD on CEBAMA reference mix and its components

As the starting material, it is important to characterise the mineralogy of the CEBAMA reference mix and its individual components. The CEBAMA mix was found to be rich in calcium- and magnesium-bearing minerals, primarily originating from its raw materials—blast furnace slag (BFS) and OPC cement (CEM I 42.5R). The other component, silica fume, is a well-known source of amorphous silica (SiO_2); therefore, XRD analysis was not conducted on this material.

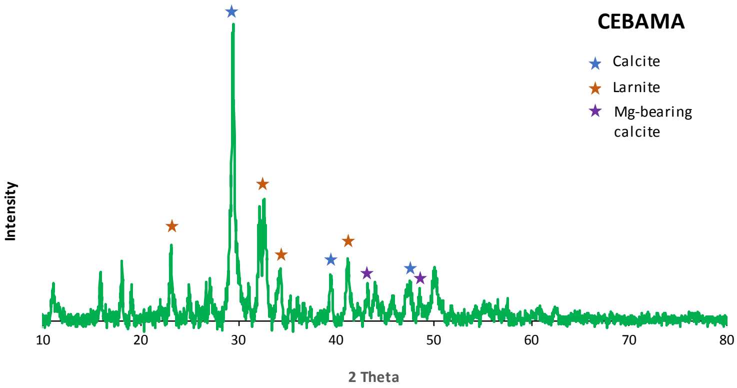

Figure S1: XRD of CEBAMA mix with the presence of calcite ($CaCO_3$), larnite (Ca_2SiO_4), and Mg-bearing calcite ($Ca_{0.94}Mg_{0.06}CO_3$).

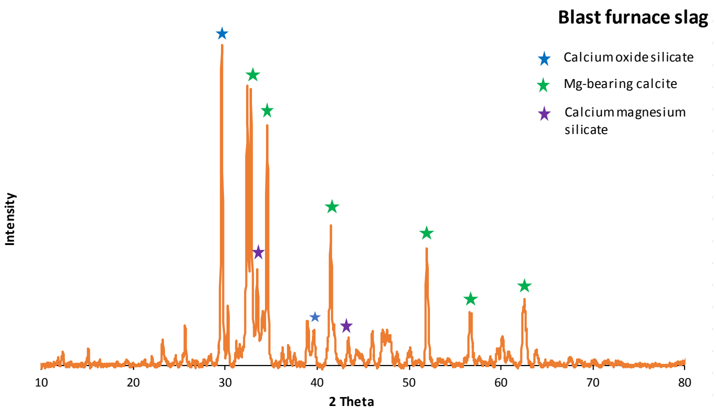

Figure S2: XRD of blast furnace slag with calcium oxide silicate, Mg-bearing calcite, and calcium magnesium silicate.

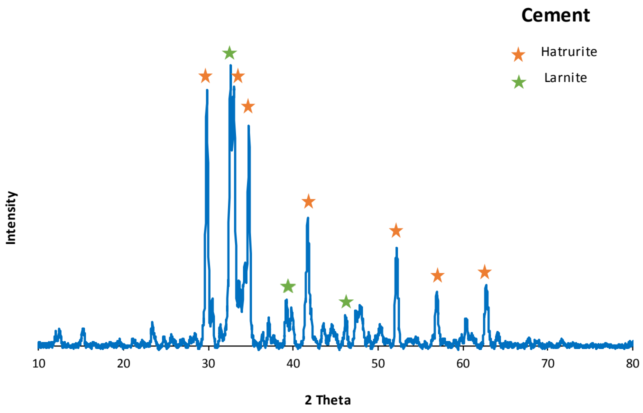

Figure S3: XRD of cement (CEM I 42.5R) showing the presence of hatrurite (Ca_3SiO_5) and larnite (Ca_2SiO_4).

2. Microcosm summary and Harpur hill sediments analysis

Since different substrates were added to each experimental system, presenting this information in a table improves clarity and helps the reader easily follow the experimental design.

Table S1: Conditions in different systems in the microcosm experiments

	Systems/Treatments	Lactate	Yeast extract	Hydrogen	Nitrate
	**No-nitrate system**				
	Groundwater				
	Groundwater + Yeast extract + Hydrogen		X	X	
	Groundwater + Lactate	X			
	**Nitrate system**				
(No-carbon)	Groundwater + Nitrate				X
(Low-carbon)	Groundwater + Yeast extract + Hydrogen + Nitrate		X	X	X
(High-carbon)	Groundwater + Lactate + Nitrate	X			X

The composition of Harpur Hill sediment used as an inoculum in this study was characterised using XRD and Ion Chromatography (IC: for anion analysis).

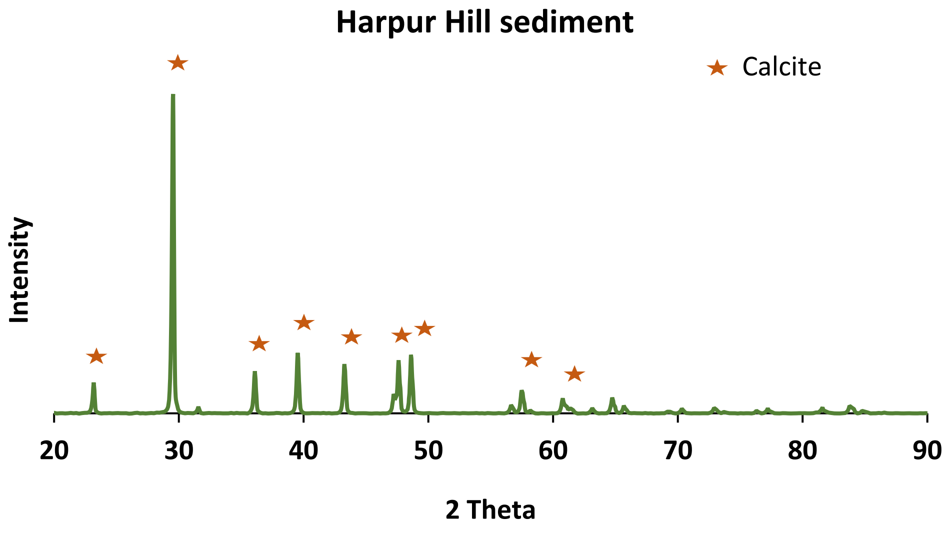

Figure S4: XRD of Harpur Hill sediment with an abundance of calcite in the sediment.

For IC sample preparation, 1g of Harpur Hill sediment was added to 10 mL of deionized water (DIW) and put in a sonicating bath for 30 minutes. After that, the solution was filtered using 0.45 µm filters. The filtered solution was further diluted 200 times in DIW and analysed using IC.

Table S2: VFAs and potential electron acceptor concentration in Harpur Hill sediment added to DIW 10% w/v.

Composition	Lactate	Acetate	Propionate	Formate	Sulphate	Nitrate	Nitrite
Amount (mM)	0.0406	0.7151	0.9609	0.0403	0.2892	0.3103	0.601

3. μ-XCT protocol

For segmenting the pores from other phases in the cement samples, the following routine was performed: The scanned images were first cropped from all three sides (x, y, z axes), and a histogram of the data then generated. A segmentation threshold was applied to the cropped images to cover all the solid phases in the cement while excluding the pores and cracks. Segmentation was performed on the resultant image to separate the pores and different phases. The grow-shrink tool was initially used to remove any small unwanted pixel features that could create ambiguity with pores. Subsequently, the global thresholding tool was applied to differentiate between the various phases. Once the phases were successfully separated, the pores were selected and locked. The pore volumes and pore diameters were quantified using the EqDiameter function, with the sieve analysis conducted on the quantified pore data.

4. PHREEQC data for the experiment

PHREEQC modelling was performed to predict the mineral phases expected to precipitate due to ion oversaturation. The results indicated that certain calcium and magnesium carbonate phases were thermodynamically favourable and could potentially form under the conditions tested.

PHREEQC input

```
SOLUTION 1
    temp      25
    pH        9.5
    pe        4
    redox     pe
    units     mmol/kgw
    density   1
    Ca        2 # 1.207 in groundwater + 0.8 mM at experimental end point, from cement
leaching (this was calculated as final, measured ICP-AES value [~ 2mM] minus 1.207)
    Cl        23.903
    Mg        3.73
    C(4)      8.528 # 0.428 in groundwater + 8.1 from lactate oxidation
    S(6)      4.997
    Na        23.764 charge
    K         0.627
    -water    1 # kg

   ------------------------------Saturation indices-------------------------------

     Phase               SI** log IAP   log K(298 K,   1 atm)

     Anhydrite        -1.53     -5.97   -4.44  Ca(SO4)
     Antarcticite    -10.63     -6.69    3.94  CaCl2:6H2O
     Aragonite         1.85     -6.46   -8.31  CaCO3
     Arcanite         -7.43     -9.28   -1.85  K2SO4
     Artinite          0.11      9.92    9.81  Mg2(CO3)(OH)2:3H2O
     Bassanite        -2.05     -5.97   -3.92  CaSO4:0.5H2O
     Bischofite      -10.82     -6.36    4.46  MgCl2:6H2O
     Bloedite         -9.13    -11.48   -2.35  Na2Mg(SO4)2:4H2O
     Brucite          -1.04     16.06   17.10  Mg(OH)2
     Burkeite        -17.24    -18.01   -0.77  Na6(CO3)(SO4)2
     C(cr)           -44.04    -76.19  -32.15  C
     Ca(cr)         -108.12    -11.27   96.85  Ca
     Ca2Cl2(OH)2:H2O(s) -17.48      9.05   26.53  Ca2Cl2(OH)2:H2O
     Ca4Cl2(OH)6:13H2O(s) -28.23     40.50   68.73  Ca4Cl2(OH)6:13H2O
     CaCl2:2H2O(cr)  -14.63     -6.68    7.95  CaCl2:2H2O
     CaCl2:4H2O(cr)  -12.04     -6.69    5.35  CaCl2:4H2O
     CaCl2:H2O(s)    -14.53     -6.68    7.85  CaCl2:H2O
     CaCO3:H2O(s)      1.14     -6.46   -7.60  CaCO3:H2O
     Calcite           2.02     -6.46   -8.48  CaCO3
     CaMg3(CO3)4(s)    5.95    -24.86  -30.81  CaMg3(CO3)4
     CaO(cr)         -16.97     15.73   32.70  CaO
     Carnallite      -15.68    -11.35    4.33  KMgCl3:6H2O
     CH4(g)          -89.14   -130.19  -41.05  CH4
     Cl2(g)          -41.39      4.59   45.98  Cl2
     CO(g)           -34.55    -49.19  -14.64  CO
     CO2(g)           -4.04    -22.19  -18.15  CO2
     Dolomite          4.53    -12.60  -17.13  CaMg(CO3)2
     Epsonite         -3.77     -5.65   -1.88  Mg(SO4):7H2O
     Gaylussite       -3.36    -12.79   -9.43  CaNa2(CO3)2:5H2O
     Glaserite       -26.07    -33.68   -7.61  Na2K6(SO4)4
     Glauberite      -13.78    -11.81    1.97  Na2Ca(SO4)2
     Gypsum           -1.36     -5.97   -4.61  CaSO4:2H2O
     H2(g)           -27.00    -27.00    0.00  H2
```

```
  H2O(g)            -1.50    -43.00  -41.50  H2O
  Halite            -4.86     -3.27    1.59  NaCl
  HCl(g)           -17.50    -11.21    6.29  HCl
  Hexahydrite       -4.01     -5.65   -1.64  Mg(SO4):6H2O
  Hydrophilite     -18.45     -6.68   11.77  CaCl2
  K(cr)            -56.78     -7.29   49.49  K
  K(OH)(s)         -18.39      6.21   24.60  K(OH)
  K-carbonate      -12.80     -9.77    3.03  K2CO3:1.5H2O
  K-trona          -14.93    -24.03   -9.10  K2NaH(CO3)2:2H2O
  K2CO3(cr)        -15.18     -9.77    5.41  K2CO3
  K2O(s)           -71.69     12.42   84.11  K2O
  Kainite          -10.45    -10.64   -0.19  KMgCl(SO4):3H2O
  Kalicinite        -5.92    -15.98  -10.06  KHCO3
  Lansfordite       -1.10     -6.14   -5.04  Mg(CO3):5H2O
  Leonhardtite      -4.76     -5.65   -0.89  MgSO4:4H2O
  Leonite          -10.95    -14.93   -3.98  K2Mg(SO4)2:4H2O
  Magnesite(nat)     2.78     -6.13   -8.91  MgCO3
  Magnesite(syn)     1.97     -6.13   -8.10  Mg(CO3)
  Mercallite       -14.09    -15.49   -1.40  KHSO4
  Mg(cr)           -90.72    -10.94   79.78  Mg
  Mg(SO4)(s)       -14.74     -5.64    9.10  Mg(SO4)
  Mg(SO4):H2O(s)    -5.52     -5.64   -0.12  Mg(SO4):H2O
  Mg-oxychlorur     -5.12     20.91   26.03  Mg2Cl(OH)3:4H2O
  Mg5(CO3)4(OH)2:4H2O(s)   1.83     -8.48  -10.31  Mg5(CO3)4(OH)2:4H2O
  MgCl2(s)         -28.38     -6.35   22.03  MgCl2
  MgCl2:2H2O(s)    -19.25     -6.35   12.90  MgCl2:2H2O
  MgCl2:4H2O(s)    -13.80     -6.36    7.44  MgCl2:4H2O
  MgCl2:H2O(s)     -22.57     -6.35   16.22  MgCl2:H2O
  Mirabilite        -4.61     -5.84   -1.23  Na2SO4:10H2O
  Na(cr)           -51.46     -5.57   45.89  Na
  Na2(CO3)(cr)      -7.45     -6.33    1.12  Na2(CO3)
  Na2CO3:7H2O(s)    -5.87     -6.33   -0.46  Na2CO3:7H2O
  Na2O(cr)         -51.59     15.87   67.46  Na2O
  Nahcolite         -3.52    -14.26  -10.74  Na(HCO3)
  Natron            -5.50     -6.33   -0.83  Na2(CO3):10H2O
  Nesquehonite      -1.04     -6.14   -5.10  Mg(CO3):3H2O
  O2(g)            -29.09    -31.99   -2.90  O2
  Pentahydrite      -4.37     -5.65   -1.28  MgSO4:5H2O
  Periclase         -5.52     16.06   21.58  MgO
  Picromerite      -10.60    -14.93   -4.33  K2Mg(SO4)2:6H2O
  Pirssonite        -3.88    -12.79   -8.91  Na2Ca(CO3)2:2H2O
  Polyhalite       -13.13    -26.87  -13.74  K2MgCa2(SO4)4:2H2O
  Portlandite       -7.08     15.73   22.81  Ca(OH)2
  Sylvite           -5.87     -5.00    0.87  KCl
  Syngenite         -7.81    -15.26   -7.45  K2Ca(SO4)2:6H2O
  Tachyhydrite     -36.78    -19.40   17.38  Mg2CaCl6:12H2O
  Thenardite        -5.48     -5.84   -0.36  Na2SO4
  Thermonatrite     -6.81     -6.33    0.48  Na2(CO3):H2O
  Trona             -9.21    -20.59  -11.38  Na3H(CO3)2:2H2O
  Vaterite           1.44     -6.46   -7.90  CaCO3

------------------
End of simulation.
------------------
```

5. FTIR analysis of carbonate deposition on cement tablets

It was challenging to scrape sufficient precipitate from the surface of the cement tablets for comprehensive mineralogical analysis. However, EDS spectra and elemental mapping confirmed that the newly deposited mineral was rich in Ca, O, and Mg. Due to the limited sample quantity, XRD analysis could not be performed. FTIR analysis was considered a suitable alternative to confirm the presence of carbonate. A very small amount of surface deposit was carefully collected and analysed using FTIR, which revealed characteristic carbonate bonding peaks. These peaks matched those of calcite ($CaCO_3$), confirming the precipitation of calcium carbonate on the tablet surface.

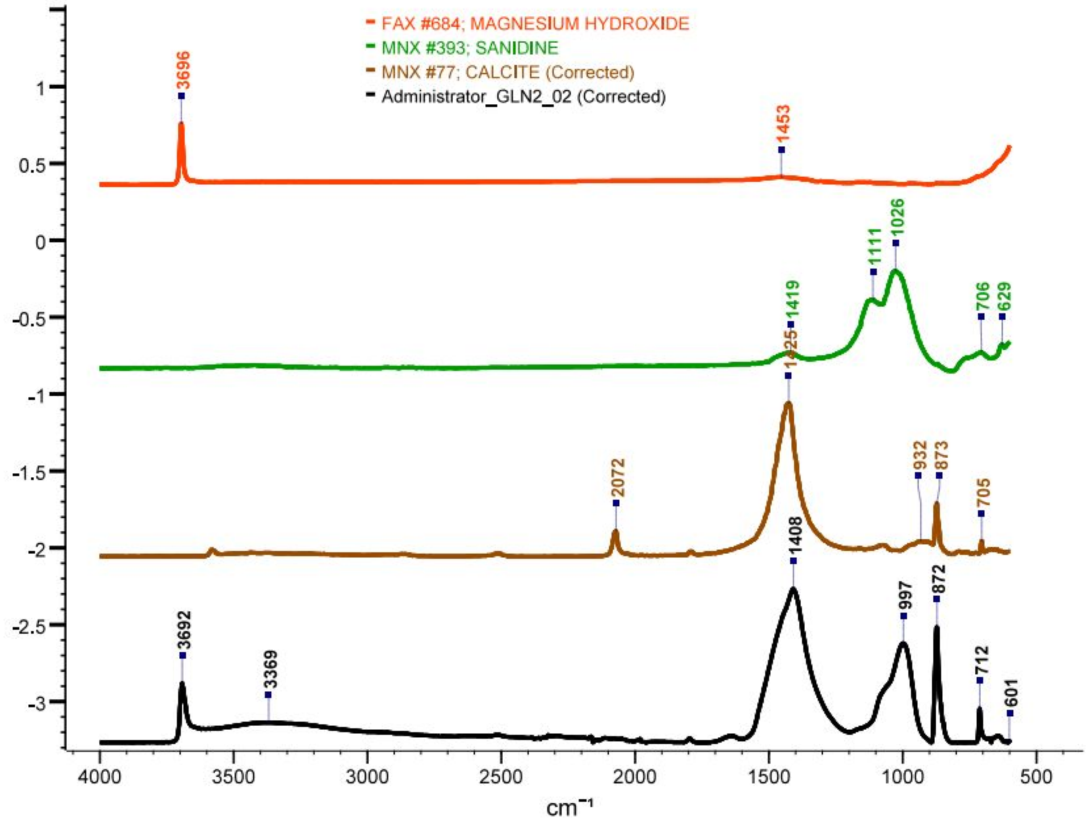

Figure S5: FTIR data from a tablet incubated in the high-carbon system, showing the presence of calcite and Magnesium hydroxide.

6. Pore count analysis

In addition to the EqDiameter vs. pore count graphs and the 3D pore visualizations across different size ranges, numerical data also support the observed reduction in pore numbers. A comparison between month 1 and month 6 for both the high-carbon and low-carbon systems clearly shows a decrease in pore count across multiple size ranges.

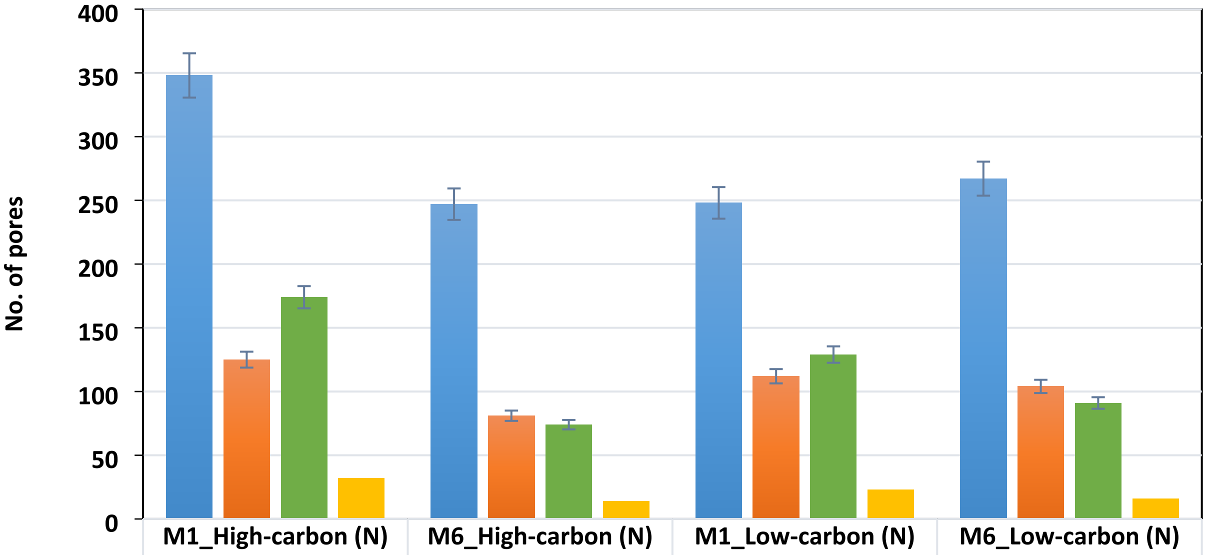

	M1_High-carbon (N)	M6_High-carbon (N)	M1_Low-carbon (N)	M6_Low-carbon (N)
100-150	348	247	248	267
150-200	125	81	112	104
200-800	174	74	129	91
400-800	32	14	23	16

Figure S6: Bar graph of the number of pores in high-carbon and low-carbon systems after one month and six months of exposure. ('N' is used to represent the system with added nitrate).

7. Microbial community data

In the experiment, Bacilli was observed to be the most dominant bacterial class after six months. To further investigate the genus-level composition within the Bacilli class, BLAST analysis was performed. This revealed that *Anaerobacillus* had become the dominant genus in the system.

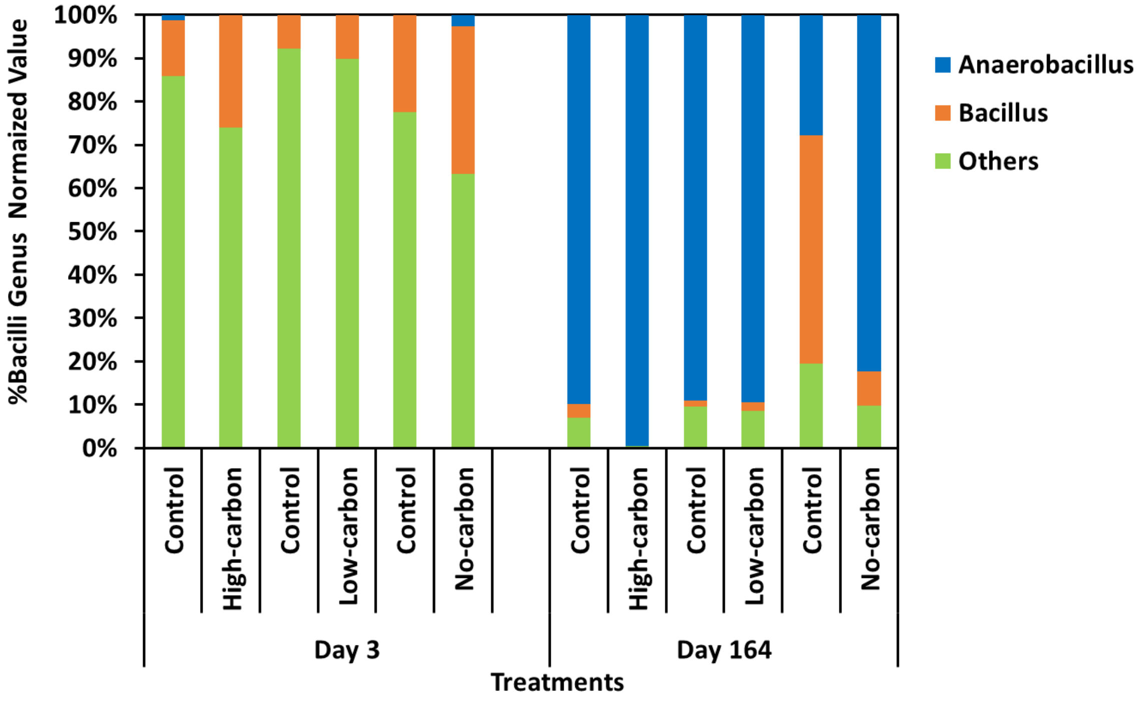

Figure S7: Data showing 16S rRNA gene sequencing for the Bacilli (% normalized value) at different stages of the experiment (Day 3; start, Day 114; middle, and Day 164; end).

8. Controls for a 6-month experiment with autoclaved Harpur Hill sediment.

To monitor the effect of Harpur hill sediment on cement tablets over 6-months without active microbes in the microcosm system. The IC (for VFAs and electron acceptor), ICP (for Ca leaching), pH, and SEM-BSE (for surface texture imaging) were as follows:

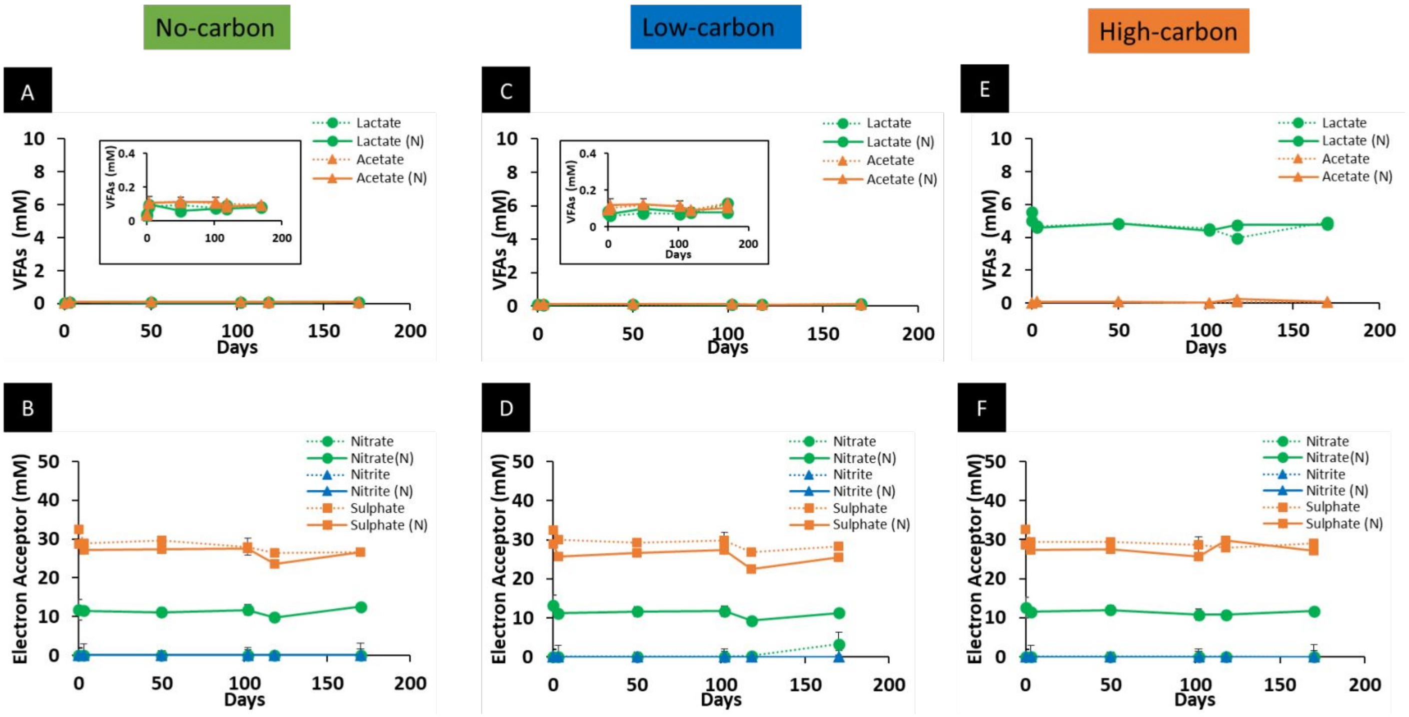

Figure S8: VFAs concentration and electron acceptor (sulfate, nitrate, nitrite) concentration for (A-B) No-added carbon, (C-D) low-carbon, (E-F) high-carbon system of microcosm. 'N' is used to represent the system with added nitrate). The dotted line (……) represents no-nitrate, and the solid line (_____) represents the nitrate system.

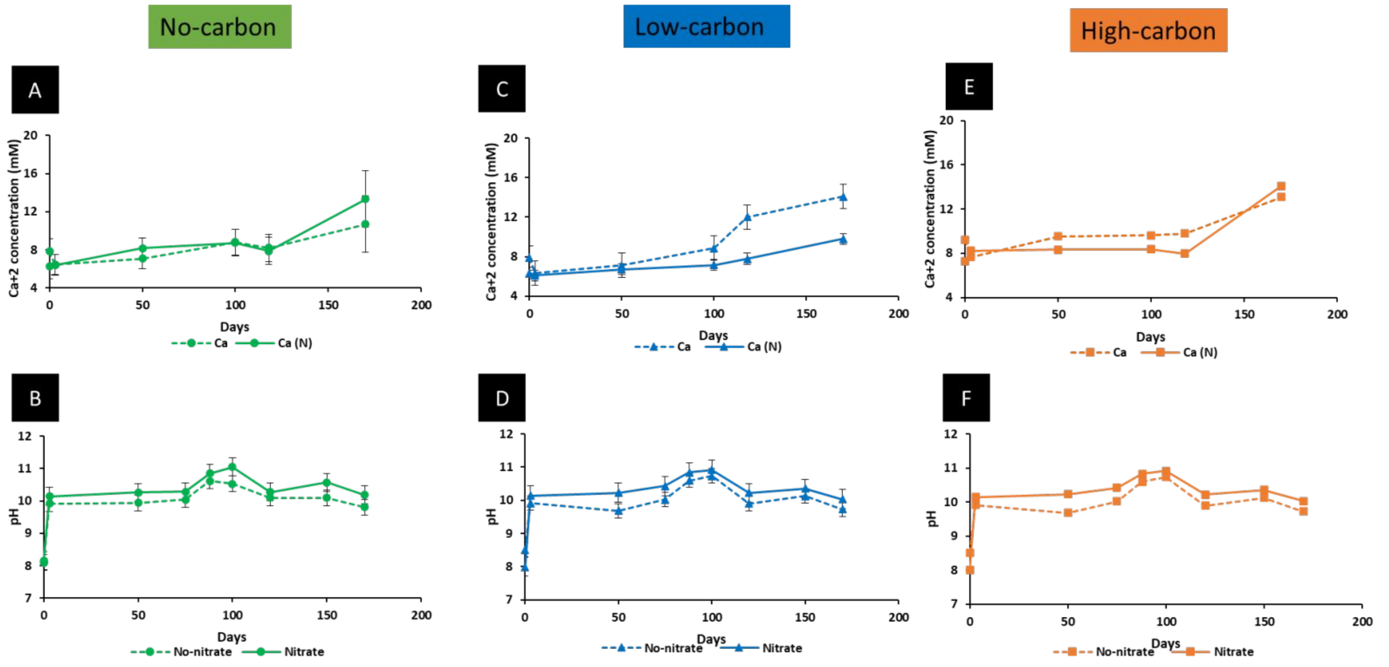

Figure S9: Calcium ion concentration and pH for autoclaved 6-month experiment system. for (A-B) No-added carbon, (C-D) low-carbon, (E-F) high-carbon system of microcosm. 'N' is used to represent the system with added nitrate). The dotted line (……) represents no-nitrate, and the solid line (_____) represents the nitrate system.

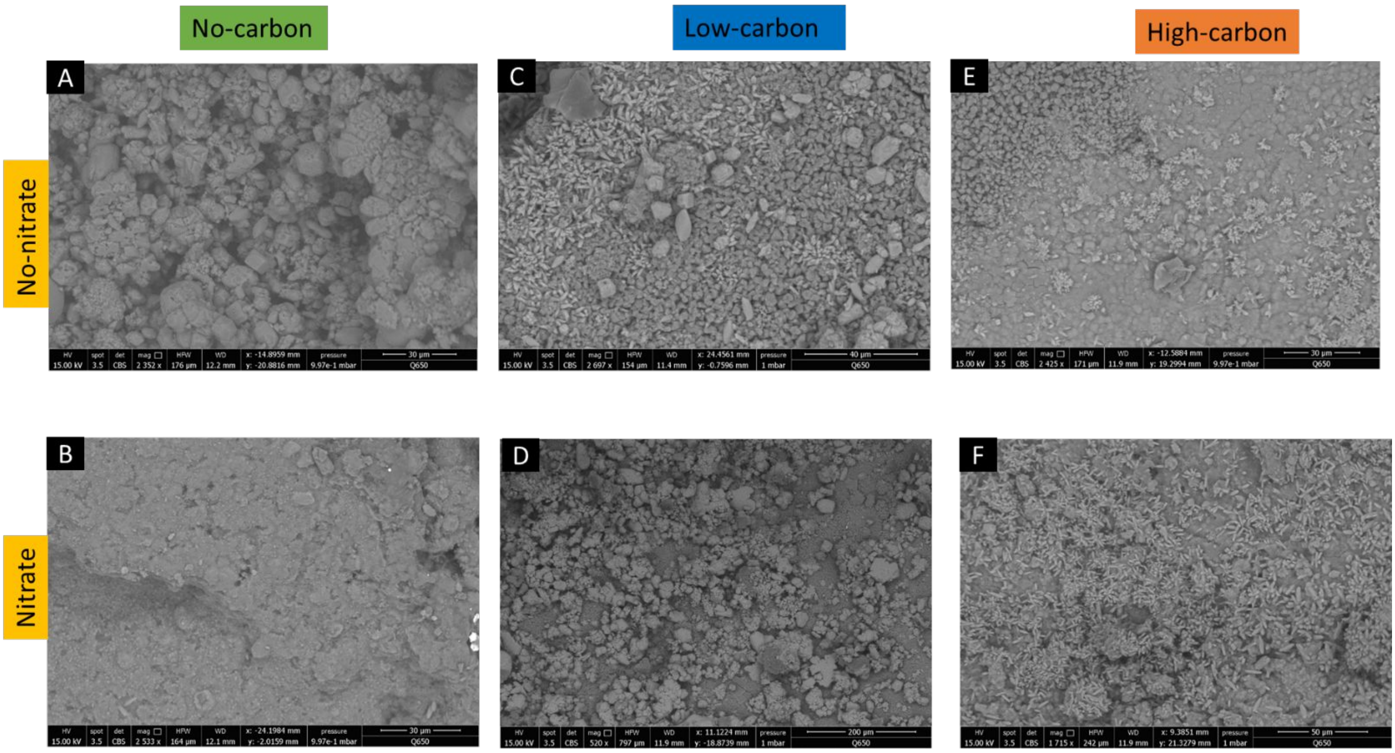

Figure S10: SEM-BSE images for comparison of one- and six-months BSE images of new minerals deposited on the surface of (A & B) No-carbon, (C & D) Low-carbon, (E & F) High-carbon. The magnification of each image is between 30 to 50 µm.

9. Controls for a 6-month experiment to monitor cement leaching.

To monitor the leaching effect of cement tablets on the experiment setup, a 6-month experiment was conducted with same setup but without harpur hill inoculum. The ICP (for Ca leaching), pH, and SEM-BSE (for surface texture imaging) were as follows:

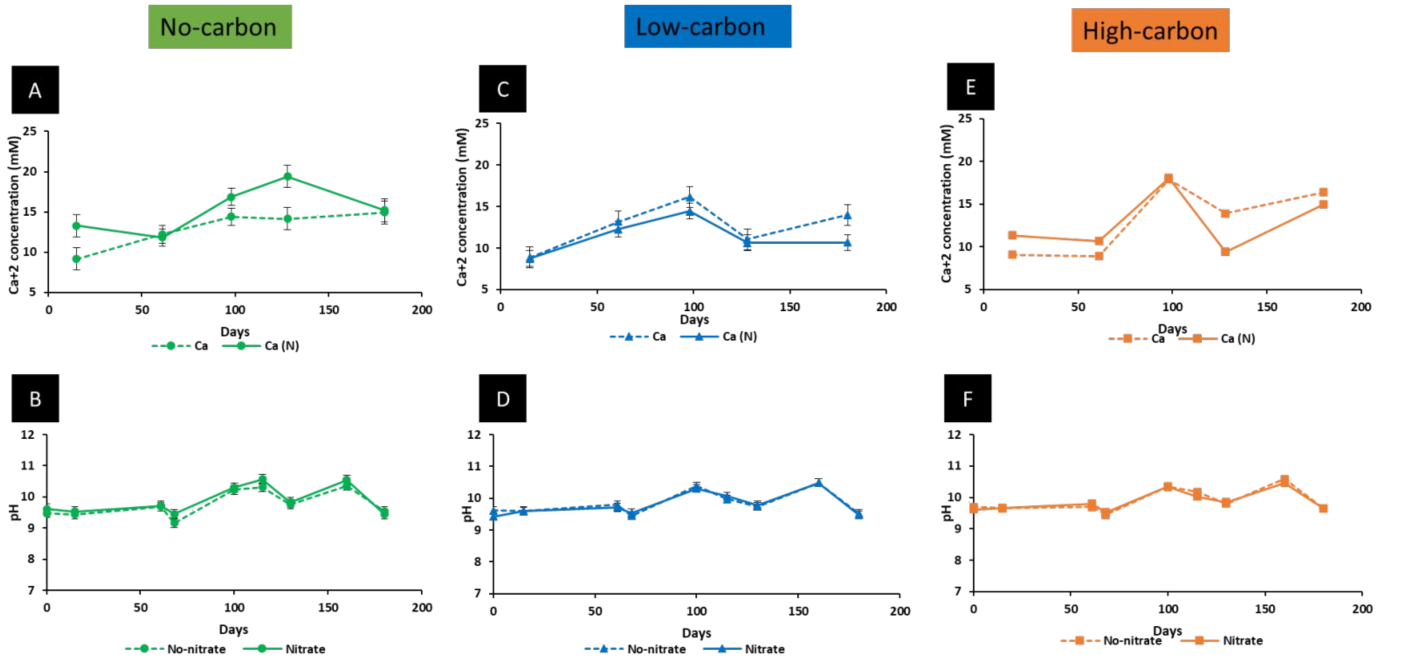

Figure S11: Calcium ion concentration and pH for autoclaved 6-month experiment system. for (A-B) No-added carbon, (C-D) low-carbon, (E-F) high-carbon system of microcosm. 'N' is used to represent the system with added nitrate). The dotted line (……) represents no-nitrate, and the solid line (______) represents the nitrate system.

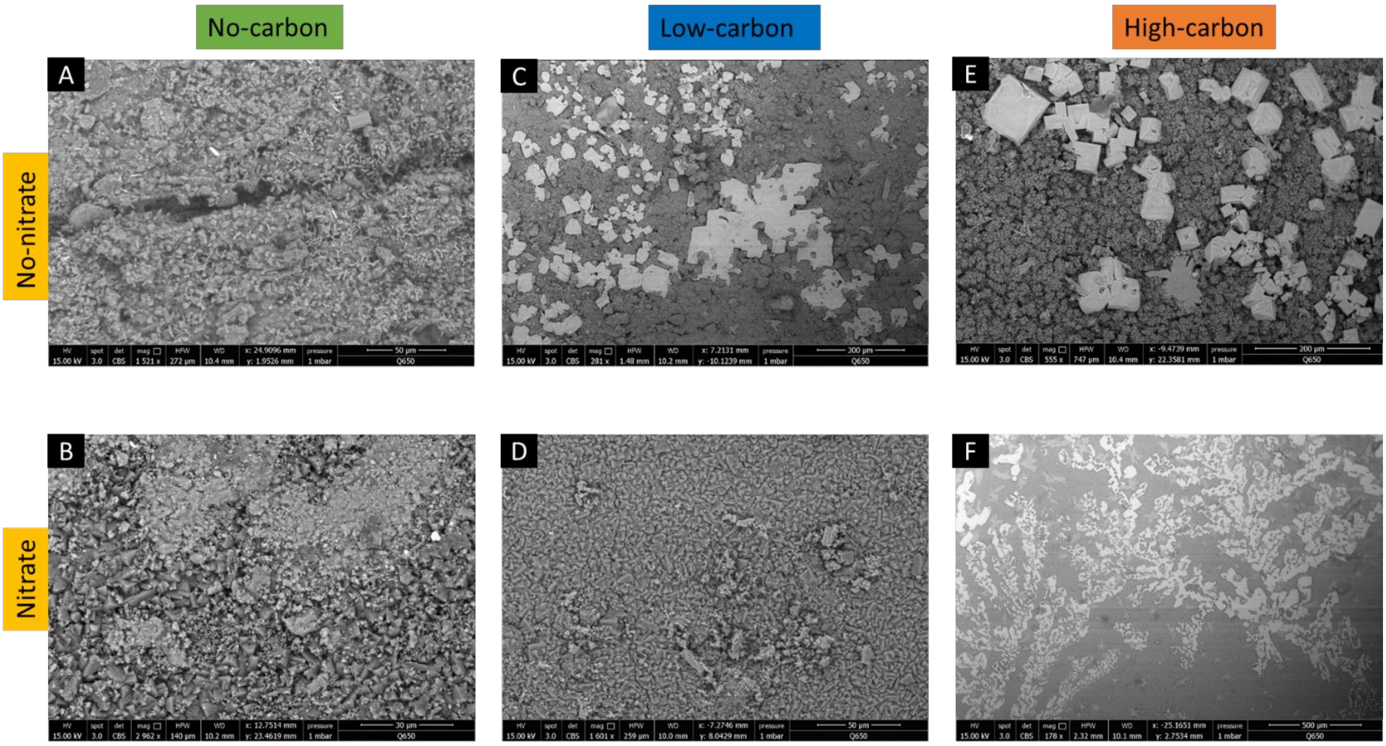

Figure S12: SEM-BSE images. Caparison of one- and six-months BSE images of new minerals deposited on the surface of (A & B) No-carbon (GN), (C & D) Low-carbon (GYN), (E & F) High-carbon (GLN). The magnification of each image is between 30 to 50 μm.
